# An examination of the psychosocial consequences experienced by children and adolescents living with congenital heart disease and their primary caregivers: a scoping review protocol

**DOI:** 10.1186/s13643-023-02249-7

**Published:** 2023-06-02

**Authors:** Tamara L. Dorfman, Mandy Archibald, Mark Haykowsky, Shannon D. Scott

**Affiliations:** 1 College of Health Sciences, Faculty of Nursing, University of Alberta, Edmonton Clinic Health Academy, University of Alberta, 3-141, 11405-87 Avenue, Edmonton, AB T6G 1C9 Canada; 2 Pediatric Cardiology, Stollery Children’s Hospital, Walter C. Mackenzie Health Sciences Centre, Unit 4C3/4C4, 8440-112 Street, Edmonton, AB T6G 2B7 Canada; 3 College of Nursing, University of Manitoba, Helen Glass Centre for Nursing, University of Manitoba (Fort Garry Campus), 89 Curry Place, Winnipeg, MB R3T 2N2 Canada

**Keywords:** Congenital heart disease, Children, Adolescents, Primary caregivers, Psychosocial outcomes, Psychosocial functioning

## Abstract

**Background:**

The chronicity of congenital heart disease (CHD) comes with significant psychosocial consequences for both children and adolescents living with CHD and their primary caregivers. Children and adolescents living with CHD undergo multiple traumatizing invasive surgical and medical procedures, struggle with disabilities resulting from their CHD, face unfair scrutiny and marginalization, and are at risk for mental health issues. Primary caregivers of children and adolescents living with CHD deal with increased stress, fear, anxiety, depression, and financial burden. The overarching objectives of this scoping review are to (1) determine the current state of knowledge on negative psychosocial consequences experienced by children and adolescents living with CHD and their primary caregivers in high-income countries and (2) inform research aimed at developing interventions in high-income countries to decrease the negative psychosocial consequences experienced by children and adolescents living with CHD and their primary caregivers.

**Methods:**

Databases and grey literature searched will include MEDLINE, CINAHL, EMBASE, PsycINFO, CENTRAL, Scopus, ProQuest Theses and Dissertations, and Google advanced search. Citation mining of included studies and relevant review articles will be completed. Studies will be screened by title and abstract and then full text by two independent reviewers, using pre-defined inclusion and exclusion criteria. Quality analysis will be conducted on all included studies by two reviewers using MMAT Version 2018. Studies will not be excluded due to quality assessment. Data from all eligible studies will be independently extracted by the two reviewers and verified by consensus. Data will be presented and synthesized in evidence tables to examine potential patterns.

**Discussion:**

The results of this review will provide recognition of the psychosocial impact of CHD and its treatments on children and adolescents living with CHD and their primary caregivers. It will also highlight interventions that have been developed to decrease these psychosocial consequences. The results from this review will inform a future integrated knowledge translation study by the first author aimed at decreasing one or more of the negative psychosocial consequences experienced by children or adolescents living with CHD and their primary caregivers.

**Systematic review registration:**

Open Science Framework (OSF) Registration, https://doi.org/10.17605/OSF.IO/ZXYGW

**Supplementary Information:**

The online version contains supplementary material available at 10.1186/s13643-023-02249-7.

## Background

### Congenital heart disease: prevalence and mortality

Approximately 1.35 million infants are born each year with congenital heart disease (CHD) [1], making CHD the most common cause of significant congenital abnormalities affecting newborn children worldwide [1–3]. Globally, birth prevalence of CHD varies between 9.3 per 1000 live births (in Asia) and 1.9 per 1000 live births (in Africa). North America and Europe fall on the higher end of this spectrum, with 6.9 per 1000 live births and 8.2 per 1000 live births, respectively [1]. These variations are likely related to limited access to health care and diagnostic facilities in low- and middle-income countries [1] and are also reflected in the mortality rates of children born with CHD worldwide [3]. Specifically, differences in mortality between industrialized versus third world countries are prominent, ranging from 3%–7% to 20%, respectively [3]. Therefore, despite CHD being the most common significant congenital abnormality, its birth prevalence and mortality varies geographically in relation to the income status of the country.

### Types of congenital heart disease and prevalence

According to the American Heart Association (AHA), at least 18 distinctive forms of CHD have been documented, many of which have additional anatomic variations [4]. These defects can be broadly classified into acyanotic CHD and cyanotic CHD. Acyanotic CHD can be further subdivided into shunt lesions (e.g., atrial septal defects (ASD), ventricular septal defects (VSD), and patent ductus arteriosus (PDA)) and obstructive lesions (e.g., aortic stenosis (AoS), pulmonary stenosis (PS), and coarctation of the aorta (CoA)). Cyanotic CHD can be further subdivided into cyanotic lesions with decreased pulmonary blood flow (e.g., tetralogy of fallot (TOF), pulmonary atresia (PA)) and cyanotic lesions with increased pulmonary blood flow (e.g., hypoplastic left heart syndrome (HLHS), transposition of the great arteries (TGA), and total anomalous venous connection (TAPVC)) [5]. The eight most common types of CHD are VSDs (2.62 per 1000 live births), ASDs (1.64 per 1000 live births), PDA (0.84 per 1000 live births), pulmonary stenosis (PS) (0.50 per 1000 live births), TOF (0.34 per 1000 live births), CoA (0.34 per 1000 live births), TGA (0.31 per 1000 live births), and AoS (0.22 per 1000 live births) [1]. Furthermore, there are significant geographical differences in the reported birth prevalence of the eight most common types of CHD. For instance, Asia, reports a higher incidence of pulmonary outflow obstructions (PS and TOF) compared to left ventricular outflow obstructions (CoA and AoS). Asia also reports a lower birth prevalence of TGA compared to Europe, North America, South America, and Oceania [1]. Therefore, CHD varies not only by subtype but also by incidence of the subtype and prevalence of common subtypes of CHD in certain parts of the world.

### Innovations in medical and surgical care of children with CHD

In high-income countries, innovations in medical and surgical care of children with CHD have significantly improved the survival rates of children born with CHD. In fact, studies in high-income countries report that 80 to 95% of children born with CHD are surviving into adulthood thereby allowing CHD to be considered a chronic rather than a life-threatening illnes*s* [6–8]. These innovations, however, mean that children living with CHD are more likely to endure multiple diagnostic tests including countless echocardiograms (ECHOs), electrocardiograms (ECGs), magnetic resonance imaging scans (MRIs) and computed tomography scans (CTs), X-rays, stress tests, and blood work. Moreover, children born with complex forms of CHD require lifelong follow-up and over 25% of children with complex CHD undergo surgeries that are palliative rather than reparative [8, 9]. As a result, several children living with CHD will endure additional cardiac surgeries and cardiac catheterizations prior to reaching adulthood and throughout the remainder of their lifetime [8]. The chronicity of CHD, therefore, means not only the possibility of multiple diagnostic tests and lifelong follow-up but also the possibility of multiple invasive and non-invasive medical and surgical interventions as a means of prolonging the lifespan of these children.

### Psychosocial consequences experienced by children and adolescents living with CHD

Importantly, lengthening the life expectancy of children born with CHD can result in significant negative psychosocial consequences related to the experience of living with a chronic and unpredictable illness [9–11]. Specifically, children and adolescents living with CHD have difficulty reconciling their illness and how it disrupts normalcy [9, 11], feel exhausted and helpless when dealing with deteriorations in their health [9], have to deal with traumatizing invasive surgical and medical procedures and disappointments related to their treatment [9], struggle with disabilities resulting from their CHD [9–11], and face unfair scrutiny and marginalization [9–11]. Additionally, when reflecting on their childhood, adults with CHD viewed their parents as being overprotective and imposing undue limitations [12]. These limitations are likely related to parents of children with CHD viewing their child as vulnerable and needing increased protection [13]. One downfall of this overprotection and restriction of activities is a risk of the child developing vulnerable children syndrome. Vulnerable children syndrome occurs when a parent perceives their child’s health issues to be bigger than their child’s actual health problem. Consequently, parents relinquish the child from their responsibilities in effort to protect them from this perceived vulnerability [14, 15]. Furthermore, the quality of life of children and adolescents living with CHD is impacted by them having a lower exercise capacity than healthy children [16, 17], impaired pulmonary function, and an increased probability of muscular deconditioning [18]. Consequently, children and adolescents with CHD do not have the same experiences as other peers their own age, are restricted in their aspirations, and are often dependent on their parents. Children and adolescents with CHD feel helpless and troubled by the debilitating physical disadvantages of their heart condition [9]. They face unpredictable complications related to their heart condition and its treatments, and they face discrimination due to their heart condition [9]. Recognition of these psychosocial consequences and interventions to decrease their impact are needed.

### Psychosocial consequences for caregivers of children and adolescents living with CHD

Additionally, families caring for children with CHD also experience negative psychosocial consequences. Negative psychosocial consequences experienced by primary caregivers of children with CHD include increased stress, fear, anxiety, depression, feelings of uncertainty, somatisation and feelings of hopelessness, and financial burden [19, 20]. There is also evidence indicating that these psychosocial morbidities can persist long term (e.g., for over 1 year) [21]. This is not surprising given that caregivers prioritize the health of their child as opposed to their own physical, mental, and emotional needs [22]. Some predictors that have been shown to influence parental distress and mental health issues in parents of children with CHD include time in relation to discharge from hospital [22], impact of their child’s illness on their daily life [22], their child’s health status [23], and if the caregivers are experiencing financial difficulties or issues with unemployment [23]. Therefore, caregivers of children with CHD need interventions that enable them to focus on their own physical and mental health and that decrease the uncertainty they deal with on a daily basis in caring for a child with CHD. They also need financial support to aid them in the care of their child.

### Purpose and rationale for the scoping review

Several studies have looked at the lived experiences of children and adolescents living with CHD [9] as well as the experiences of their caregivers [19]. One systematic review has been completed looking at quantitative assessments of psychosocial adjustment and health-related quality of life in children and adolescents following open heart surgery for repair of a congenital heart defect [24]. Similarly, one systematic review and meta-analysis has been completed focusing on quantitative psychosocial measurements of behavior, self-esteem, and social cognition in children and adolescents with severe CHD [25]. Another systematic review has been completed looking at quantitative assessments of emotional and behavioral regulation and social development of infants and children (from birth to 6 years and 11 months) who had heart surgery for CHD in the early stages of their life [26]. Additionally, an integrative review of qualitative studies has been completed looking at parents’ perception of emerging adults with CHD [13].

However, a knowledge synthesis including qualitative, quantitative, multi-method, and mixed methods study designs is yet to be completed mapping the current state of knowledge and research activity with regard to the negative psychosocial consequences experienced by children and adolescents living with CHD and their primary caregivers in high-income countries. A synthesis of interventions aimed at decreasing these consequences for these two populations in high-income countries has also not been completed. Determining the current state of knowledge on an existing topic and overall research activity in a specific area in addition to identifying research priorities are key indications for doing a scoping review [27, 28]. Therefore, a scoping review will be completed with the overarching goals of (1) determining the current state of knowledge on negative psychosocial consequences experienced by children and adolescents living with CHD and their primary caregivers in high-income countries, and (2) informing research aimed at developing interventions in high-income countries to decrease the negative psychosocial consequences experienced by children and adolescents living with CHD and their primary caregivers.

### Objectives of the scoping review

The specific objectives for this scoping review were developed using the mnemonic PCC (Population, Concept, and Context) (see Additional file 1) [29]. These objectives are to (1) determine what negative psychosocial consequences have been experienced by children and adolescents living with CHD and their primary caregivers in high-income countries; (2) determine what factors contribute to the development of the negative psychosocial consequences experienced by children and adolescents living with CHD and their primary caregivers in high-income countries; (3) identify current and past interventions aimed at decreasing the negative psychosocial consequences experienced by children and adolescents living with CHD and their primary caregivers in high-income countries; (4) map the interventions developed in high-income countries to their intended populations (i.e., children and adolescents living with CHD or their primary caregivers), subgroups within these two populations, and the psychosocial consequences they aim to impact; and (5) identify gaps in knowledge and research priorities.

## Registration

This review has been registered with Open Science Framework (OSF DOI is https://doi.org/10.17605/OSF.IO/ZXYGW). This scoping review protocol is aligned with the PRISMA-ScR guidelines [30] (see Additional file 2).

## Inclusion/exclusion criteria

### Study participants

Studies will be included in this scoping review if they were conducted on children or adolescents aged 0 to 19 years living with CHD or their primary caregivers. The age cut-off for the review is based on the World Health Organization’s (WHO) definition of adolescence [31]. Studies that involved broader age groups will be included provided that they have subgroup data for age 0 to 19 years. Studies that have adults, with CHD, retrospectively reflect on their experiences growing up with CHD will not be included as the adults’ perception of their experiences may differ from the perceptions they had as a child or an adolescent and this is not the focus of the review. Parents’ or caregivers’ perceptions of their child’s experiences and the negative psychosocial consequences they experience as a result of their heart condition and its treatments will be included as the child may not be old enough or have the cognitive capacity to report on their own experiences. In addition, some studies may compare the child or adolescents’ perception to that of their parent or caregiver, which could be an interesting comparator.

CHD will be defined as a defect or abnormality within the heart itself or blood vessels near the heart that is present at birth. Studies conducted on children or adolescents with acquired heart disease (meaning they were not born with the heart disease) or children with an inherited or acquired arrhythmia will not be included in the review. Studies conducted on children with multiorgan syndromes present at birth will also not be included in the review. Studies conducted on children or adolescents with CHD and children and adolescents with acquired heart disease will be included if subgroup data for the different types of heart diseases is provided. Similarly, studies conducted on children and adolescents with an inherited or acquired arrhythmia and children and adolescents with CHD will be included provided that subgroup data for the different conditions is provided. Studies conducted on children and adolescents with CHD and children or adolescents with multiorgan syndromes will be included provided that subgroup data for the different conditions is provided.

Primary caregiver will be defined as a non-medical person that is primarily responsible for the care of a child or adolescent living with CHD. Studies conducted on parents, guardians, foster parents, older siblings, and grandparents will be included as long as they were one of the child or adolescents’ primary caregivers. Studies conducted on healthcare workers (e.g., physicians, nurses, respiratory therapists, health care aides, and respite workers) and non-primary caregivers (e.g., extended family, younger siblings, daycare workers, and babysitters) will not be included. Similarly, studies conducted on caregivers of children or adolescents with acquired heart disease, inherited or acquired arrhythmias, or multiorgan syndromes will be excluded. Studies conducted on primary caregivers of children or adolescents with CHD and primary caregivers of children and adolescents with acquired heart disease will be included if subgroup data for the different types of heart diseases is provided. Similarly, studies conducted on primary caregivers of children and adolescents with an inherited or acquired arrhythmia and primary caregivers of children and adolescents with CHD will be included provided that subgroup data for the different conditions is provided. Studies conducted on primary caregivers of children and adolescents with CHD and primary caregivers of children or adolescents with multiorgan syndromes will be included provided that subgroup data for the different conditions is provided. Studies conducted on primary caregivers and non-primary caregivers of children and adolescents living with CHD will be included provided that subgroup data for the different types of caregivers is provided. Prenatal studies conducted on caregivers expecting a child with CHD will also be excluded. Studies conducted on primary caregivers expecting a child with CHD and primary caregivers of a child or adolescent living with CHD will be included provided that subgroup data is available for the two different caregiver groups.

### Concept

For the purpose of this review, psychosocial health will be defined as a “changing condition that involves the reciprocal adjustment and dependency between an individual and their social environment” ([32],p.6). Components of psychosocial health include individual aspects (i.e., physical or biological characteristics of the individual, experiences or perceptions, psychological or mental processes, and behavior and lifestyle), an individual’s social environment (i.e., social relationships, social networks, and social structures), an individual’s spirituality, and the availability of resources and support [32]. This review specifically examines negative psychosocial consequences, factors that contribute to the development of these negative psychosocial consequences, and interventions to decrease the occurrence of negative psychosocial consequences in children and adolescents living with CHD and their primary caregivers. Importantly, for the purpose of this review, living with CHD will include not only the consequences of the heart disease itself but also the treatments the child or adolescent receives and their potential complications. Therefore, studies that examine at least one negative psychosocial consequence (e.g., negative emotions, negative feelings, cognitive and emotional processes, negative behavior, negative health behaviors, poor lifestyle, negative body image, poor self-esteem, negative impact on social relationships or networks, negative experiences with social structures, material and financial disadvantages, negative impact on spirituality, impaired development, or adjustment); one or more factors contributing to the development of a negative psychosocial consequence(s); or an intervention developed with the goal of decreasing a negative psychosocial consequence(s) will be included in this review.

### Context

For the purpose of this review, the context will be limited to high-income countries because they have more money and resources available to care for children and adolescents with CHD as compared to low- and middle-income countries [33]. Consequently, the negative psychosocial consequences experienced by children and adolescents living with CHD and their primary caregivers in high-income countries are likely to differ from those living in middle- and low-income countries due to the increased availability of money and resources to support both their care and their families. High-income countries will be defined using the World Bank definition which states high income countries are those whose gross national income (GNI) per capita was $12,696 or more in 2020 [34]. The high-income countries identified by the World Bank as having a GNI per capita of $12,696 [34] are listed in Additional file 3. Studies that take place in both high-income and middle-income countries or in both high-income and low-income countries will only be included if subgroup data is provided for the participants from the different countries.

### Types of evidence sources

The evidence sources that will be included in this review are completed primary quantitative, qualitative, multi-method, and mixed methods research studies. Secondary analyses of primary research data and unpublished theses or dissertations on quantitative, qualitative, multi-method, and mixed methods research studies will also be included. For the purpose of this review, a study will be determined to be multi-method when it uses both qualitative and quantitative methods or data but does not integrate the findings from the two methods [35]. For the purpose of this review, a study will be defined as mixed methods if it uses both quantitative and qualitative methods and meaningfully integrates the two data forms at an applicable stage of the research (e.g., sampling; analysis) [35]. Study protocols will not be included in the review as they are not completed research. Conference abstracts and posters of unpublished primary research studies will not be included in the review due to inadequate resources and time for finding relevant conference abstracts and posters. Furthermore, abstracts and posters typically contain inadequate information to assess the design, methods, risk of bias, outcomes, and the results of the study [36]. Other evidence sources that will be excluded in this review are published abstracts, case reports, all types of knowledge synthesis reviews, letters, commentaries, websites, opinion pieces, blogs, magazines, pamphlets, clinical guidelines, books, book chapters, scientific statements or reports, association or group statements or reports, and social media groups and posts.

### Other exclusion criteria

Studies not reported in English will be excluded due to the cost and time involved in translating the material. Studies reported prior to the year 2000 will be excluded due to the remarkable advances in all aspects of pediatric cardiovascular medicine and surgery in the last 50 years of the twentieth century. These advances include changes in cardiopulmonary bypass, surgical techniques and equipment, cardiac catheterization techniques and equipment, pharmacological therapies used in the treatment of CHD, and the training of pediatric cardiologists and pediatric cardiac surgeons [37]. These advances have allowed CHD to become a chronic disease as opposed to a life-threatening disease. Additionally, the structure of families and how lay people access healthcare and healthcare information has changed in the last 20 years especially with the internet and worldwide web going public in the mid 1990s and becoming indispensable to the majority of families in high-income countries by the mid 2000s [38]. Therefore, it is possible that the negative psychosocial consequences experienced by children and adolescents living with CHD and their primary caregivers and the factors contributing to the development of these consequences have changed since prior to the year 2000. The types of interventions available to decrease these consequences have likely changed as well.

See Table 1 for a summary of the inclusion and exclusion criteria and Additional file 4 for the screening form that will be used in this review. The screening form was trialed independently by two reviewers and adapted based on feedback from both reviewers.

**Table 1:** Inclusion and exclusion criteria

Screening criteria	Inclusion criteria	Exclusion criteria
Language study is reported in	▪ English	▪ Not English
Year study was published	▪ Published after 1999	▪ Published before 1999
Study participants	▪ Human studies ▪ Children and adolescents *(aged 0 to 19 years)* born with CHD^a^ ▪ Parents, guardians, foster parents, older siblings, or grandparents *primarily responsible* for the care of children or adolescents born *(age 0 to 19 years)* with CHD, including those that ask their perceptions of their child’s experiences^a^	▪ Animal studies ▪* Only* includes Adults *(age 20 years and older)* born with CHD ▪ Ages of participants are *NOT* reported ▪ Adults with CHD who *retrospectively reflect* on their experiences growing up with CHD ▪* Only* children, adolescents, and/or adults with acquired heart disease ▪* Only* children, adolescents, or adults with inherited and/or acquired heart arrythmias ▪* Only* children, adolescents, and/or adolescents with multi-organ syndromes present at birth ▪* Only* primary caregivers of adults (age 20 + years) born with CHD ▪* Only* non-primary caregivers of children adolescents, or adults living with CHD ▪* Only* healthcare providers ▪* Only* caregivers of children, adolescents and/or adults with acquired heart disease ▪* Only* caregivers of children, adolescents, and/or adults with inherited or acquired arrhythmias ▪* Only* caregivers of children, adolescents, and/or adults with multi-organ syndromes present at birth ▪* Only prenatal studies* conducted on caregivers expecting a child with CHD
Study outcomes reported	▪* At least one* negative psychosocial consequence (e.g., negative emotions, negative feelings, cognitive and emotional processes, negative behavior, negative health behaviors, poor lifestyle, negative body image, poor self-esteem, negative impact on social relationships or networks, negative experiences with social structures, material and financial disadvantages, negative impact on spirituality, impaired development, or adjustment) ▪ Examines factors contributing to the development of negative psychosocial consequence(s) ▪ Reports on an intervention developed to decrease negative psychosocial consequence(s)	▪* Does NOT* examine negative psychosocial consequences, factors that contribute to negative psychosocial consequences, or interventions with the goals of decreasing negative psychosocial consequences
Country where the study takes place	▪ High-income country (GNI per capita of $12,696)^b^	▪* Only* low- or middle- income country^c^
Evidence source	▪ Primary quantitative, qualitative, multi-method, and mixed method studies ▪ Secondary analysis of primary data ▪ Theses and dissertations	▪ Conference/meeting abstracts or posters ▪ Published abstracts ▪ Case reports ▪ Knowledge syntheses/reviews ▪ Study protocols ▪ Letters ▪ Commentaries ▪ Websites ▪ Opinion pieces ▪ Blogs ▪ Magazines ▪ Pamphlets ▪ Clinical guidelines ▪ Scientific statements or reports ▪ Association or group statements or reports ▪ Books ▪ Book chapters ▪ Social media groups and posts

*CHD* congenital heart disease (i.e., born with a defect or abnormality in the heart or blood vessels near the heart; includes both complex and simple forms of CHD)

^a^Note: studies which include other participants but provide subgroup data on the participants of interest will be included

^b^See Additional file 3 for list of high-income countries as per The World Bank Group

Note: studies will be included if subgroup data is listed for high-income countries when the study takes place in both high-income and low-or middle- income countries

^c^Not listed in Additional file 3

## Search strategy

The search strategy for this review was developed in consultation with a research librarian from the University of Alberta (U of A). Medical subject headings (MeSH) and free text keywords and phrases were developed based on synonyms and terms related to CHD (including specific types of CHD), child or adolescent, non-medical caregiver, and attributes of psychosocial health. The databases that will be searched will include MEDLINE via OVID, CINAHL via EBSCOhost, EMBASE via OVID, PsycINFO via OVID, Cochrane’s Central Register of Controlled Trials (CENTRAL), and Scopus. MEDLINE, EMBASE, CINHAL, and Scopus will be used as they index a number of medical and health science journals. PsycINFO will be used because it includes international literature on psychology which may provide important articles on the emotional processes and mental health for the two populations examined in this review. All databases including CENTRAL will be used to find articles on clinical interventions with the goal of reducing negative psychosocial consequences in children and adolescents with CHD and their primary caregivers. The English language and published date search filters will be used based on the review’s inclusion criteria. Filters to look for search terms specifically in the abstract and title only will be used in OVID databases, and filters to look for search terms in the title, abstract, and keywords only in CENTRAL will be used as this is recommended by the librarian. Filters to limit literature sources in EMBASE will be used as they are recommended by the librarian. The search strategies for the above databases can be found in Additional file 5. In order to limit publication bias, grey literature will be searched using the ProQuest Theses and Dissertations Global database and Google. The advanced Google search function will be used to focus the search to the specific subject area of the review. File type will be limited to pdfs, PowerPoints (.ppt), or word documents (.doc). Only the first 100 results from each search will be evaluated for inclusion in the review as this represents the most relevant results well still being a feasible amount to screen based on the time and resources available [39]. Four unique search strategies will be applied using the advanced Google search function. The search strategies for ProQuest Theses and Dissertations Global and Google can be found in Additional file 6. Lastly, both backward and forward citation mining will be used to identify articles missed through database searching that may meet requirements for inclusion in this review. First, backward citation mining will be completed by hand searches of reference lists of all included studies and all relevant systematic reviews found during database and grey literature searching. Then forward citation mining of all included studies will be completed using the Web of Science citation tracking analysis to identify newly published articles that cite included studies in this review and that may have been missed through initial database and grey literature searching. Web of Science was chosen as its citation tracking is accurate, reproducible, and more likely to be used by formal organizations for this purpose.

## Data management

EndNote 20 will be used for reference citation management of the studies identified through database and grey literature searching. EndNote 20 was chosen as citations can be efficiently uploaded into it from database searches and PDF full texts can be attached to their citations and stored within it. Additionally, studies identified by grey literature searches can be uploaded if available in RIS format or manually entered. Furthermore, EndNote 20 has both desktop and online applications that make it easily accessible on multiple computers and by all members of the study team through shared online EndNote libraries [40]. In addition to EndNote, Covidence web-based systematic review manager will be used for screening of studies identified through database searching (including ProQuest Theses and Dissertations Global) at both the title/abstract and full-text stages. Covidence was chosen because it is compatible with EndNote. Covidence organizes the citations, removes duplicates, and walks the reviewers through the screening stages of the review in a manner that is streamlined and efficient. It also allows for storage of full-text articles [41]. Microsoft excel will also be used to track the records identified through database searching as they move through the different stages of the scoping review. Godin’s et al. (2015) strategy will be applied to managing grey literature. Article records found through advanced Google search will be “bookmarked” in Google Chrome at the time the search is completed and filed into categorized folders based on the four unique search strategies applied. The filing of bookmarks will be utilized in effort to provide the reviewers with easy access to the bookmarked websites using Google Chrome’s Bookmark Manager [39]. Bookmarking the records during screening will also help prevent the same record from being identified repeatedly throughout the search and screening process [39]. Microsoft Excel will also be used to track studies identified by searching Google. Information tracked in Microsoft Excel will include date of search, search terms used, Uniform Resource Locator (URL), total number of records, number of results screened, potentially relevant records, and number of records included in the review.

## Screening of studies for inclusion in the review

In stage 1 of screening, the studies identified through database searches (including ProQuest Theses and Dissertations Global database) will be screened by title and abstract independently by two reviewers using the above pre-defined inclusion and exclusion criteria. If no abstract is available, a portion of the text will be obtained to facilitate screening at this stage. All studies will be evaluated as include, exclude, or unclear. In stage 2 of screening, studies evaluated as include or unclear will have their full texts retrieved for independent screening by the two reviewers against the same pre-defined inclusion and exclusion criteria. Studies will be evaluated as include or exclude. Studies that meet all the pre-defined inclusion criteria will be included in the review.

In stage 3 of screening, records identified through Google advanced search will be screened, independently, by two reviewers against the above pre-defined inclusion and exclusion criteria by title and the short text below the title. All studies will be evaluated as include, exclude, or unclear. Relevant records, marked as include or unclear, will be put forward for independent screening of their full texts by the two reviewers against the same above pre-defined exclusion and inclusion criteria. Studies will be evaluated as include or exclude. Studies that meet all the pre-defined inclusion criteria will be included in the review.

In stage 4 of screening, additional studies identified through forward and backward citation mining will be screened independently by two reviewers by title and abstract using the above pre-defined inclusion and exclusion criteria. If no abstract is available, a portion of the text will be obtained to facilitate screening at this stage. All studies will be evaluated as include, exclude, or unclear. The full text of all studies that were labeled as include or unclear will be independently screened against the same pre-defined inclusion and exclusion criteria. Studies will be evaluated as include or exclude. Studies that meet all the pre-defined inclusion criteria will be included in the review.

At all stages of screening, disagreements between the reviewers will be resolved through discussion and, when necessary, the involvement of a third reviewer. All peer-reviewed studies included in the review will proceed to the quality appraisal and data extraction phases of the review. Non-peer-reviewed studies will proceed to the data extraction phase of the review only.

## Quality appraisal

The quality of all full-text peer-reviewed studies included in this review will be appraised independently by two reviewers using the Mixed Methods Appraisal Tool (MMAT) Version 2018 (see Additional file 7) [42]. MMAT was chosen as it allows for the appraisal of qualitative, quantitative, and mixed method studies using the same tool [42]. It has also undergone recent evaluations to inform its usefulness and content validity [43, 44]. Additionally, a user guide is available for the latest version ensuring the reviewers have clear guidance on how to use the tool [45]. At the start of quality analysis, the included peer-reviewed studies will be organized into the five categories described in the MMAT Version 18 tool (qualitative, quantitative randomized control trials, quantitative non-randomized control trials, quantitative descriptive, and mixed methods). Multi-method studies will be grouped into a sixth category. This sixth category will be made as the MMAT Version 2018 tool does not clearly state how to complete quality appraisal for multi-method study designs. Therefore, for the purpose of this review, multi-method studies will be appraised using both the question set for the qualitative MMAT section and the most applicable quantitative design MMAT question set. Once categorized, the tool will be piloted on six studies (one for each of the categories outlined above if they are available from the peer-reviewed studies included in the review) by both reviewers independently and compared at the start of the quality appraisal process to ensure both reviewers understand how to use the tool. Once quality appraisal is completed by both reviewers on all the peer-reviewed studies included in the review than the two reviewers will compare their appraisals to identify disagreements. Disagreements will be resolved through discussion and when needed the involvement of a third reviewer. MMAT Version 2018 discourages calculating an overall quality score from the ratings of each of the criterion of the scale [42]. Therefore, each criterion will be reported in a table for each peer-reviewed included study. Instead, a sensitivity analysis will be completed and described contrasting the quality of the studies in each of the categories. The quality of included peer-reviewed studies will not be compared across categories. Non-peer-reviewed studies, including theses and dissertations, obtained through grey literature searching will not undergo quality appraisal as there is currently no tool available for assessment of their quality. Studies will not be excluded from data synthesis based on their quality, but the results from these studies will be interpreted with caution. Furthermore, if only poor-quality studies are available for a specific population or subgroup then this will be considered as a reason for further research into this area.

## Data extraction

The data extraction form developed for this scoping review and instructions for use of this form can be found in Additional file 8. This form will be independently piloted by two data extractors on five included studies and then reviewed through discussion to determine if any adaptations need to be made prior to extracting data from the remainder of the studies. If there are disagreements on adaptations to the data extraction form, which cannot be resolved through discussion, a third data extractor will be involved.

The data extraction form consists of six sections. The data extracted for section one of the form includes citation information for the study, study design characteristics, and information about study participants. Citation information includes the title of the manuscript, author details, journal published, year published, volume and issue of the publication journal, page numbers of the manuscript, funding sources, and other potential publication biases. Study design information includes region or country of the study, the study setting, the type of study design, study methodology, study eligibility criteria, recruitment and sampling procedures, and enrollment start and end dates for the study. It also includes information related to the length of follow-up and allocation to intervention and comparison groups which is specific to intervention studies only. Information to be extracted about study participants includes study population type (i.e., children or adolescents living with CHD or their caregivers), age of participants, gender distribution of participants, types of CHD of study participants or types of CHD the caregiver’s child has, other participant co-morbidities or the caregiver’s child’s co-morbidities, and other not already recorded participant characteristics.

Section two of the data extraction form will be filled out if the study describes negative psychosocial consequence(s) experienced by children or adolescents living with CHD and/or their primary caregivers in high-income countries. If the study describes factors that influence the development of psychosocial consequences experienced by children or adolescents living with CHD and/or their primary caregivers in high-income countries, then section three of the form will be completed. In both these sections, data extractors will check a box that describes the study design and complete the corresponding study design (i.e., quantitative design or qualitative design) table (Additional file 8). If the study is a multi-method or mixed methods design both the quantitative and qualitative design tables will be filled out. In section two, the quantitative supporting data to be collected includes the psychosocial consequence measured, psychosocial scale used in the study (if one was used), and results from the study. In section two, the qualitative supporting data to be collected includes themes illustrating psychosocial consequences and brief descriptions of the themes. In section three, the quantitative supporting data to be collected includes identifying factors that influence the development of the negative psychosocial consequence and quantitative results that support those factors. In section three, the qualitative data to be collected includes themes illustrating factors that influence the development of negative psychosocial consequences and brief descriptions of the themes.

Section four will be filled out by data extractors if the study describes an intervention designed to decrease a negative psychosocial consequence(s) experienced by children or adolescents living with CHD and/or their primary caregivers in high-income countries (e.g., an intervention designed to increase resilience in the child or their parent/caregiver). Information to be extracted in this section includes who the intervention was designed for, who was involved in designing the intervention, a description of the intervention, what psychosocial consequence(s) it is designed to decrease, if the intervention is effective, how the intervention is measured, adverse effects of the intervention, and any other comments about the intervention that the extractor feels is important. Data extracted in section five will include if there was any missing data and how the missing data was handled, whether study participants withdrew or were lost to follow-up, and why they may have withdrawn or were lost to follow-up. Data extraction in section six includes miscellaneous data such as key conclusions from the study authors, limitations discussed by the authors, if correspondence is required with the study authors and why, and other comments that the data extractor may have.

Data extraction will take place using electronic data extraction forms in Microsoft Word. Two extractors will independently complete the form for data extraction on each of the included studies. If multiple reports are identified from the same study, the data from all reports will be extracted into a single data extraction form. Once extraction is completed by both the extractors, the data extracted will be compared to ensure agreement or to identify discrepancies. One data extractor’s electronic data collection form will be used to record changes after consensus is reached by the two extractors in a different ink color. Disagreements will be resolved by discussion between the two extractors. If disagreements cannot be resolved, a third person may be brought in to help settle the disagreement. Any agreement that cannot be resolved will be addressed by contacting the study authors, if the study authors cannot be reached, the disagreement(s) will be reported in the review.

## Data synthesis

Studies included in the review will be grouped into tables based on study design and populations investigated (i.e., children or adolescents living with CHD or their primary caregivers). The studies grouped as children and adolescents living with CHD will be further sub-grouped by age, types of CHD, and country or region of the study. Studies grouped in terms of primary caregivers will be further sub-grouped by type of caregiver, type of CHD the primary caregiver’s child has, and country or region of the study. These groupings will allow for a narrative account of the range, type, and distribution of the studies included in the scoping review [27]. Next, the studies will be grouped thematically in terms of the objectives of the scoping review (see objectives section above) they describe. Lastly, interventions will be mapped back to their intended populations (i.e., children and adolescents living with CHD or their primary caregivers), subgroups within these two populations, and the psychosocial consequences they aim to impact in effort to identify which specific negative psychosocial consequences are lacking an intervention. The data will be organized thematically, and the evidence tables will be analyzed to identify knowledge gaps in the literature and where further research is needed.

## Discussion

This scoping review has been developed to be a comprehensive map of the current state of knowledge and research activity with regard to the negative psychosocial consequences experienced by children and adolescents living with CHD and their primary caregivers in high-income countries. It has the potential to impact both the care and education of children and adolescents living with CHD and their primary caregivers in several ways. First, the results of this study will both synthesize and recognize the psychosocial impact of CHD and its treatments on children and adolescents living with CHD and their primary caregivers in high-income countries. Second, it will identify factors that contribute to the development of these negative psychosocial consequences in children and adolescents living with CHD and their primary caregivers in high-income countries, which may be used as a starting point for the development of interventions to decrease these negative psychosocial consequences in these two populations. Third, the results of the review will highlight interventions that have been developed with the goal of decreasing these negative psychosocial consequences. Fourth, it will identify gaps where interventions have not been developed to address specific negative psychosocial consequences experienced by these children and adolescents living with CHD and their primary caregivers in high-income countries. Lastly, the results from this review will inform a future integrated knowledge translation (KT) study, developed by the first author of the review, aimed at developing a KT intervention for parents or primary caregivers of children or adolescents living with CHD that is designed to decrease one or more of the negative psychosocial consequences experienced by children or adolescents living with CHD and their primary caregivers in high-income countries.

## Supplementary Information

**Additional file 1. **Application of the PCC mnemonic.

**Additional file 2. **Preferred reporting items for systematic reviews and meta-analyses extension for scoping reviews (Prisma-ScR) checklist.List of high-income countries based on the world bank definition.

**Additional file 3. **List of high-income countries based on the world bank definition.

**Additional file 4. **Scoping review: Eligibility and screening criteria form.

**Additional file 5. **Search strategies for library databases.

**Additional file 6. **Search strategies for grey literature.

**Additional file 7. **Mixed Methods Appraisal Tool (MMAT), Version 2018.

**Additional file 8.** Instructions for completing data extraction form and sample data extraction form.

## Data Availability

Not applicable.

## Abbreviations

AoS: Aortic stenosis
AHA: American Heart Association
ASD: Atrial septal defect
CENTRAL: Cochrane’s Central Register of Controlled Trials
CHD: Congenital heart disease
CoA: Coarctation of the aorta
CTs: Computed tomography scans
.doc: Document
ECHOs: Echocardiograms
ECGs: Electrocardiograms
GNI: Gross national income
HLHS: Hypoplastic left heart syndrome
KT: Knowledge translation
MeSH: Medical subject heading
MMAT: Mixed Methods Appraisal Tool
MRIs: Magnetic resonance imaging scans
OSF: Open Science Framework
PA: Pulmonary atresia
PCC: Population, concept, context
PDA: Patent ductus arteriosus
.ppt: PowerPoint
PRISMA-ScR: Preferred Reporting Items for Systematic Reviews and Meta-Analyses extension for Scoping Reviews
PS: Pulmonary stenosis
TAPVC: Total anomalous pulmonary venous connection
TGA: Transposition of the great arteries
TOF: Tetralogy of Fallot
U of A: University of Alberta
URL: Uniform Resource Locator
VSD: Ventricular septal defect
WHO: World Health Organization
