# Correction: An examination of the psychosocial consequences experienced by children and adolescents living with congenital heart disease and their primary caregivers: a scoping review protocol

**DOI:** 10.1186/s13643-023-02271-9

**Published:** 2023-06-20

**Authors:** Tamara L. Dorfman, Mandy Archibald, Mark Haykowsky, Shannon D. Scott

**Affiliations:** 1 College of Health Sciences, Faculty of Nursing, University of Alberta, Edmonton Clinic Health Academy, University of Alberta, 3‑141, 11405‑87 Avenue, Edmonton, AB T6G 1C9 Canada; 2 Pediatric Cardiology, Stollery Children’s Hospital, Walter C. Mackenzie Health Sciences Centre, Unit 4C3/4C4, 8440‑112 Street, Edmonton, AB T6G 2B7 Canada; 3 College of Nursing, University of Manitoba, Helen Glass Centre for Nursing, University of Manitoba (Fort Garry Campus), 89 Curry Place, Winnipeg, MB R3T 2N2 Canada

**Correction: Syst Rev 12, 90 (2023)**10.1186/s13643-023-02249-7

Following publication of the original article [1], the authors’ identified errors on the assignment of affiliations.

The author group section above shows the correct assignment and the original article [1] has been corrected.
