# Supplementary material for: An examination of the psychosocial consequences experienced by children and adolescents living with congenital heart disease and their primary caregivers: a scoping review protocol
Source: Syst Rev. 2023 Jun 2;12:90. doi: 10.1186/s13643-023-02249-7 (PMC10239103; doi:10.1186/s13643-023-02249-7)
Supplement: Supplementary file 1 — Additional file 1. Application of the PCC mnemonic. [file 13643_2023_2249_MOESM1_ESM.docx]

**Additional file 1**

**Application of the PCC mnemonic**

| **Population** | Children and adolescents living with CHD **OR** primary caregivers of children and adolescents living with CHD |
| --- | --- |
| **Concept** | Primary Concept: negative psychosocial consequences  Secondary Concept: factors contributing to the development of these negative psychosocial concepts  Secondary Concept: interventions developed with a goal of reducing these negative psychosocial consequences |
| **Context** | High income countries as defined by the World Bank definition. |
