# Supplementary material for: An examination of the psychosocial consequences experienced by children and adolescents living with congenital heart disease and their primary caregivers: a scoping review protocol
Source: Syst Rev. 2023 Jun 2;12:90. doi: 10.1186/s13643-023-02249-7 (PMC10239103; doi:10.1186/s13643-023-02249-7)
Supplement: Supplementary file 3 — Additional file 3. List of high-income countries based on the world bank definition. [file 13643_2023_2249_MOESM3_ESM.docx]

**Additional file 3**

**List of high-income countries based on the world bank definition**

| ANDORRA  ANTIGUA AND BARBUDA  ARUBA  AUSTRALIA  AUSTRIA  The BAHAMAS  BAHRAIN  BARBADOS  BELGIUM  BERMUDA  BRITISH VIRGIN ISLANDS  BRUNEI DARUSSALAM  CANADA  CAYMAN ISLANDS  CHANNEL ISLANDS  CHILE  CROATIA  CURACAO  CYPRUS  CZECH REPUBLIC  DENMARK  ESTONIA  FAROE ISLANDS  FINLAND  FRANCE  FRENCH POLYNESIA  GERMANY  GIBRALTAR  GREECE  GREENLAND  GUAM | HONG KONG SAR, CHINA  HUNGARY  ICELAND  IRELAND  ISLE OF MAN  ISRAEL  ITALY  JAPAN  KOREA, REP.  KUWAIT  LATVIA  LIECHTENSTEIN  LITHUANIA  LUXEMBOURG  MACAO SAR, CHINA  MALTA  MONACO  NAURU  NETHERLANDS  NEW CALEDONIA  NEW ZEALAND  NORTHERN MARIANA ISLANDS  NORWAY  OMAN  PALAU  POLAND  PORTUGAL  PUERTO RICO  QATAR  SAN MARINO  SAUDI ARABIA  SEYCHELLES | SINGAPORE  SINT MAARTEN (DUTCH PART)  SLOVAK REPUBLIC  SLOVENIA  SPAIN  ST. KITTS AND NEVIS  ST. MARTIN (FRENCH PART)  SWEDEN  SWITZERLAND  TRINIDAD AND TOBAGO  TURKS AND CAICOS ISLANDS  UNITED ARAB EMIRATES  UNITED KINGDOM  UNITED STATES  URUGUAY  VIRGIN ISLANDS (U.S.) |
| --- | --- | --- |

The World Bank Group (2022). *High income*. Retrieved May 6, 2022 from <https://data.worldbank.org/country/XD>
