# Supplementary material for: An examination of the psychosocial consequences experienced by children and adolescents living with congenital heart disease and their primary caregivers: a scoping review protocol
Source: Syst Rev. 2023 Jun 2;12:90. doi: 10.1186/s13643-023-02249-7 (PMC10239103; doi:10.1186/s13643-023-02249-7)
Supplement: Supplementary file 4 — Additional file 4. Scoping review: Eligibility and screening criteria form. [file 13643_2023_2249_MOESM4_ESM.docx]

**Additional file 4**

**Scoping review: Eligibility and screening criteria form**

| **Research Questions and Search Parameters** | | |
| --- | --- | --- |
| **Research Questions:**  *Question # 1a:* What are the negative psychosocial consequences experienced by children and adolescents living with CHD and/or their primary caregivers in high-income countries?  *Question #1b:* What factors contribute to the development of these negative psychosocial consequences in children and adolescents living with CHD and/or their primary caregivers in high-income countries?  Question # 2*:* What interventions have been developed in high-income countries with the goal of decreasing the negative psychosocial consequences experienced by children and adolescents living with CHD and/or their primary caregivers?  *Question # 3a:* What knowledge gaps exist? (i.e., in what groups are interventions needed (e.g., age group of the children or adolescents), for which caregivers (e.g., mothers, fathers, foster parents, grandparents), subtype of CHD, what negative psychosocial consequences are lacking interventions, etc.)  *Question # 3b:* What are the research priorities? | | |
| **Restrictions:** published in English; published in the year 2000 or later, high-income countries | | |
| **Data Sources:**   - *Databases:* 1. MEDLINE via OVID, CINAHL via EBSCOhost, EMBASE via OVID, PsycINFO via OVID, Cochrane Central Register of Controlled Trials (CENTRAL), and Scopus - *Grey Literature:* ProQuest Theses and Dissertations Global and Google (advanced search function will be used in Google, file type will be limited to pdfs, PowerPoints (.ppt), or word documents (.doc), and only the first 100 results) - *Other:* Scanning the reference list of included articles and all relevant knowledge synthesis reviews (backward citation tracking), forward citation tracking using Web of Science | | |
| **Filters used:** English Language, publish date, limit search to abstract and title (. ti,ab.) in Ovid databases, limit to abstract, title, and keywords (:ti,ab,kw) in CENTRAL, literature source filters will be used in EMBASE | | |
| **Abbreviations/ Definitions:** CHD= Congenital Heart Disease  Definition of CHD = a defect or abnormality in the heart or blood vessels near the heart; includes both complex and simple forms of CHD) | | |
| **Screening Question** | **Inclusion Criteria** | **Exclusion Criteria** |
| 1. Is the study reported in English?   **Yes /No/ Unclear** | - Reported in English | - Not reported in English |
| 1. Is the study published in the year 2000 or later?   **Yes /No/ Unclear** | - Published after 1999 | - Not published after 1999 |
| 1. Does this study involve children or adolescents with congenital heart disease **OR** a non-medical primary caregiver of a child or adolescent with congenital heart disease?   **Yes /No/ Unclear** | - Human Studies - Studies conducted on children or adolescents aged 0 to 19 years of age **born with CHD.** - Studies conducted on parents, guardians, foster parents, older siblings, or grandparents **primarily responsible** for the care of children or adolescents (age 0 to 19 years) with CHD, including those that ask their perceptions of their child’s experiences. - Studies on **both** children and adolescents with CHD and with inherited or acquired heart arrythmias **that provided subgroup data.** - Studies conducted on **both** children and adolescents with congenital heart disease and acquired heart disease **that provided subgroup data** - Studies on **both** children and adolescents with congenital heart disease and multiorgan syndromes **that provide subgroup data.** - Studies on **both** primary caregivers of children and adolescents with congenital heart disease and inherited or acquired heart arrythmias **that provided subgroup data.** - Studies conducted on **both** primary caregivers of children and adolescents with congenital heart disease and acquired heart disease **that provided subgroup data.** - Studies on **both** primary caregivers of children and adolescents with congenital heart disease and multiorgan syndromes **that provide subgroup data.** - Studies conducted on **both** primary caregivers expecting a child with CHD and primary caregivers of a child or adolescent living with CHD **that provide subgroup data.** | - Animal Studies - Only includes Adults (**age 20 years and older)** born with CHD. - Age of participants are **NOT** reported. - Studies that have adults, with CHD, **retrospectively reflect** on their experiences growing up with CHD. - Studies conducted **only** on children, adolescents, and/or adults with acquired heart disease. - Studies conducted **only** on children, adolescents, or adults with inherited and/or acquired heart arrythmias. - Studies conducted **only** on children, adolescents, and/or adolescents with multi-organ syndromes present at birth. - Studies conducted **only** on primary caregivers of adults (aged 20+ years) born with CHD. - Studies conducted **only** on Non-primary caregivers of children adolescents, or adults living with CHD (e.g., extended family, daycare care or day home workers, babysitters, etc.) - Studies conducted **only** on Healthcare providers (e.g., physicians, nurses, respiratory therapists, healthcare aides, respite workers) - Studies conducted **only** on caregivers of children, adolescents and/or adults with acquired heart disease. - Studies conducted **only** on caregivers of children, adolescents, and/or adults with inherited or acquired arrhythmias. - Studies conducted **only** on caregivers of children, adolescents, and/or adults with multi-organ syndromes present at birth. - **Only Prenatal Studies** conducted on caregivers expecting a child with CHD. |
| 1. Does the study report on at least one negative psychosocial consequence **AND/OR** one or more factors contributing to the development of a negative psychosocial consequence **AND/OR** an intervention developed with the goal of decreasing a negative psychosocial consequence?   **Yes /No/ Unclear** | - The study examines **at least one** negative psychosocial consequence (e.g., negative emotions, negative feelings, cognitive and emotional processes, negative behavior, negative health behaviors, poor lifestyle, negative body image, poor self-esteem, negative impact on social relationships or networks, negative experiences with social structures, material and financial disadvantages, negative impact on spirituality, impaired development, or adjustment). - The study examines factors contributing to the development of negative psychosocial consequence(s). - The study reports on an intervention developed to decrease negative psychosocial consequence(s). | - The study **does not** examine negative psychosocial consequences. - The study **does not** examine factors contributing to the development of negative psychosocial consequences. - The study **does not** describe an intervention developed with the goal of decreasing negative psychosocial consequences. |
| 1. Does the study take place in a high-income country based on the world bank definition? | - The study **takes place** in a high-income country (GNI per capita of $12,696).   **See Additional file 3** for list of High-Income Countries as per The World Bank Group.   - Studies that take place both in high-income and middle-income countries where **subgroup data is provided for the countries**. - Studies that take place in both high-income and low-income countries where **subgroup data is provided for the countries.** | - The study **takes place** in a low-or middle- income country. - Studies that take place both in high-income and middle-income countries where **subgroup data is not provided for the countries**. - Studies that take place in both high-income and low-income countries where **subgroup data is not provided for the countries.**   **Note:** the country will not be listed in Additional file 3 |
| 1. Is the type of evidence source one of the following?   **Yes /No/ Unclear** | - Primary quantitative, qualitative, multi-method, and mixed method studies - Secondary analysis of primary data - Theses and dissertations | - Conference/Meeting abstracts or posters - Published abstracts - Case reports - Knowledge syntheses/reviews - Study protocols - Letters - Commentaries - Websites - Opinion pieces - Blogs - Magazines - Pamphlets - Clinical guidelines - Scientific statements or reports - Association or group statements or reports - Books - Book chapters - Social media groups and posts |
| **Final Decision:** | | |
| **Include** | **Exclude** | **Unclear** |
| **If unclear, further action taken:** | | |
|  | | |
| **If excluded reason for exclusion:** | | |
|  | | |
| **Other comments:** | | |
|  | | |
