# Supplementary material for: An examination of the psychosocial consequences experienced by children and adolescents living with congenital heart disease and their primary caregivers: a scoping review protocol
Source: Syst Rev. 2023 Jun 2;12:90. doi: 10.1186/s13643-023-02249-7 (PMC10239103; doi:10.1186/s13643-023-02249-7)
Supplement: Supplementary file 5 — Additional file 5. Search strategies for library databases. [file 13643_2023_2249_MOESM5_ESM.docx]

**Additional file 5**

**Search strategies for library databases**

**Medline Search Strategy**

1 exp adolescent/ or exp child/ or exp infant/ or exp minor/ or exp Pediatrics/

2 (child* or adolesc* or teen* or preteen* or pre-teen* or tween* or juvenile* or youth* or minor* or kid* or "school age" or schoolchild* or "school child*" or toddler* or infan* or neonat* or baby or babies or newborn* or "new born*" or p?ediatric* or prepubesc* or pre-pubesc* or pubescen*).ti,ab.

3 exp caregivers/ or exp grandparents/ or exp parents/ or exp Legal Guardians/ or exp Siblings/

4 (caregiver* or "care giver" or carer* or caretaker* or "care taker*" or guardian* or parent* or stepparent or mom* or mother* or father* or Dad* or stepfather* or stepmother* or grandparent* or grandm* or grandpa* or grandfather* or sibling* or sister* or brother*).ti,ab.

5 heart defects, congenital/ or exp aortic coarctation/ or exp aortico-ventricular tunnel/ or exp arrhythmogenic right ventricular dysplasia/ or exp bicuspid aortic valve disease/ or exp cor triatriatum/ or exp anomalous left coronary artery/ or dextrocardia/ or exp ductus arteriosus, patent/ or exp ebstein anomaly/ or exp ectopia cordis/ or exp eisenmenger complex/ or exp heart septal defects/ or exp hypoplastic left heart syndrome/ or exp "isolated noncompaction of the ventricular myocardium"/ or exp quadricuspid aortic valve/ or exp "tetralogy of fallot"/ or exp "transposition of great vessels"/ or exp tricuspid atresia/ or "trilogy of fallot"/ or exp univentricular heart/ or persistent left superior vena cava/ or exp pulmonary atresia/ or exp scimitar syndrome/ or exp vascular ring/ or exp cardiomyopathy, dilated/ or exp cardiomyopathy, hypertrophic/ or exp cardiomyopathy, restrictive/ or exp aortic valve disease/ or exp heart valve prolapse/ or exp mitral valve insufficiency/ or exp mitral valve stenosis/ or exp pulmonary valve insufficiency/ or exp pulmonary valve stenosis/ or exp tricuspid valve insufficiency/ or exp tricuspid valve stenosis/ or exp aortic valve stenosis/ or exp Truncus Arteriosus/ or exp Fontan Procedure/

6 ("congenital heart disease" or "congenital heart defect*" or "cyanotic heart disease" or "cyanotic heart defect*" or "p?ediatric heart disease" or "aortic valve stenosis" or "aortic atresia" or "quadricuspid aortic valve" or "bicuspid aortic valve" or "atrial septal defect*" or "aortic coarctation" or "coarctation of the aorta" or "hypoplastic aortic arch" or "interrupted aortic arch" or "atrioventricular canal" or "atrioventricular tunnel" or "atrioventricular septal defect" or "dextro transposition of the great arteries" or "levo transposition of the great arteries" or "transposition of the great arteries" or "transposition of the great vessels" or "Ebstein* anomaly" or "patent ductus arteriosus" or "patent foramen ovale" or "pulmonary valve stenosis" or "Tetralogy of Fallot" or "Trilogy of Fallot" or "Truncus Arteriosus" or hemitruncus or "single ventricle" or "univentricular heart" or "anomalous pulmonary venous" or "Scimitar syndrome" or "pulmonary venous stenosis" or "ventricular septal defect*" or "hypoplastic left heart syndrome" or "pulmonary atresia" or "pulmonary stenosis" or "tricuspid atresia" or "tricuspid stenosis" or "double outlet right ventricle" or "double inlet left ventricle" or "mitral atresia" or "mitral stenosis" or "congenital cardiomyopathy" or "p?ediatric cardiomyopathy" or "dilated cardiomyopathy" or "restricted cardiomyopathy" or " hypertrophic cardiomyopathy" or "arrhythmogenic right ventricular dysplasia" or "mitochondrial noncompaction" or "mitral valve disease" or "absent pulmonary valve" or "hypoplastic right heart syndrome" or Fontan or "vascular ring" or "Cor Triatriatum" or "pulmonary artery stenosis" or "pulmonary artery sling" or "double aortic arch" or "absent heart valve" or "Shone* complex*" or "aortic insufficiency" or "tricuspid insufficiency" or "mitral insufficiency" or "pulmonary insufficiency" or "pulmonary regurgitation" or "tricuspid regurgitation" or "mitral regurgitation" or "aortic regurgitation" or dextrocardia or "crisscross heart" or "Eisenmenger complex" or "Eisenmenger Syndrome" or "Ectopia Cordis" or "atrial isomerism*").ti,ab.

7 exp psychosocial Intervention/ or exp psychosocial functioning/ or exp psychosocial support systems/

8 (psychosocial or psycho-social).ti,ab.

9 exp adaptation, psychological/ or attitude/ or exp attitude to death/ or exp attitude to health/ or exp catastrophization/ or exp optimism/ or exp pessimism/ or exp respect/ or exp stereotyping/ or exp "wit and humor as topic"/ or exp behavior/ or exp child rearing/ or exp defense mechanisms/ or exp emotions/ or exp human development/ or exp mental competency/ or exp motivation/ or exp neurobehavioral manifestations/ or exp personality/ or exp psychology, social/ or exp temperance/ or exp psychological phenomena/ or exp mental disorders/ or exp physical endurance/ or exp physical exertion/ or exp physical fitness/ or exp Fatigue/ or exp alcoholism/ or exp substance abuse, intravenous/ or exp substance abuse, oral/

10 ("lived experience*" or "live through" or attitude* or psych* or mental* or "mental process*" or "mental health" or "mental disorder*" or mind* or feel* or thought* or emotion* or "social cognition*" or "cognitive process*" or depression or behavio?r* or anxiety or anxious or "attention deficit" or alcoholi* or lifestyle or suicid* or "cognitive deficit*" or "self-esteem" or "self-concept" or "body image" or "body perception" or fear* or "stress disorder" or anger or distrust or "medical trauma" or "physical trauma" or addict* or panic or "sexual activit*" or "sex life" or "sex drive" or "drug use" or "drug abuse*" or grief or griev* or bereave* or wellness or wellbeing or well-being or "physical health" or "physical shape" or "physical condition*" or "physical fitness*" or endurance or fatigue or "exercise tolerance" or "coping skill*" or "coping strateg*").ti,ab.

11 exp social justice/ or exp public opinion/ or social problems/ or exp adverse childhood experiences/ or exp bullying/ or exp dangerous behavior/ or divorce/ or exp emotional abuse/ or exp poverty/ or exp social segregation/ or exp suicide/ or exp underage drinking/ or exp social structure/ or social welfare/ or exp community resources/ or exp culture/ or exp "ethnic and racial minorities"/ or exp family relations/ or exp family separation/ or exp hierarchy, social/ or exp medicalization/ or exp minority groups/ or exp secularism/ or exp social capital/ or exp social conditions/ or exp social environment/ or exp social isolation/ or exp social marginalization/ or exp social norms/ or exp social vulnerability/ or exp sociodemographic factors/ or exp socioeconomic factors/ or exp needs assessment/ or exp return to school/ or exp culturally competent care/ or exp "delivery of health care, integrated"/ or exp health services accessibility/ or exp healthcare disparities/ or exp health inequities/ or exp educational status/

12 (friendship* or "family relation*" or "personal relationship*" or "interpersonal relationship*" or "social environment*" or "social factor*" or "social barrier*" or "social inclusion" or "social network*" or employer* or "social group*" or "social relationship*" or "professional relationship*" or "intimate relationship*" or "sexual partner" or "marital status" or "social structure*" or "society as a whole" or "socioeconomic status" or "political system*" or "judicial system*" or "legal system*" or "health* system*" or "health care system*" or "healthcare system*" or "health* program*" or "health care program*" or "healthcare group*" or "health care group*" or "education system*" or "school system" or "cultural environment*" or patriarchy or marginali* or discriminat* or judgement or "social isolation" or prejudice* or bias* or bigotry or intolerance* or "narrow mindedness" or inequalit* or inequit* or disadvantage* or disempower* or empower* or stigma* or vulnerable or bullying or bullied or oppress* or persecute* or "traumatic event*" or workplace* or "work place* or social justice" or "social injustice*").ti,ab.

13 employment/ or exp employment, supported/ or exp unemployment/ or exp workplace/ or exp income/

14 (finance* or income or "finance* service*" or "employ* service*" or "finance* resource* " or "employ* resource*" or "material resource*" or employ* or unemploy* or job? or "distribution of material resource*" or "social* disadvantage*" or "neo material" or money or "pecuniary fund*" or bankrupt* or welfare or "socioeconomic factor*").ti,ab.

15 exp religion/ or exp "religion and psychology"/ or spirituality/

16 (spiritual* or religion* or religious* or Ignatian or otherworldliness or unearthliness or incorporeality or piousness or devoutness or holiness or religiosity or pietism or piety or reverence or transcendence or "relationship to the transcendent" or "religious affiliation*").ti,ab.

17 exp Developed Countries/

18 ("high income countr*" or "high-income countr*" or "high income nation*" or "high-income nation*" or "developed countr*" or "developed nation*" or "first world countr*" or "first world nation*").mp. [mp=title, abstract, original title, name of substance word, subject heading word, floating sub-heading word, keyword heading word, organism supplementary concept word, protocol supplementary concept word, rare disease supplementary concept word, unique identifier, synonyms]

19 exp aruba/ or exp curacao/ or exp sint maarten/ or north america/ or exp canada/ or exp greenland/ or exp united states/ or chile/ or exp uruguay/ or exp brunei/ or exp singapore/ or exp bahrain/ or exp israel/ or exp kuwait/ or exp oman/ or exp qatar/ or exp saudi arabia/ or exp united arab emirates/ or exp hong kong/ or exp macau/ or exp japan/ or exp korea/ or exp taiwan/ or europe/ or exp andorra/ or exp austria/ or exp belgium/ or exp estonia/ or exp latvia/ or exp lithuania/ or exp croatia/ or exp czech republic/ or exp hungary/ or exp poland/ or exp slovakia/ or exp slovenia/ or exp france/ or exp germany/ or exp gibraltar/ or exp united kingdom/ or exp channel islands/ or exp england/ or exp northern ireland/ or exp greece/ or exp ireland/ or exp italy/ or exp liechtenstein/ or exp luxembourg/ or exp monaco/ or exp netherlands/ or exp portugal/ or exp san marino/ or exp denmark/ or exp finland/ or exp iceland/ or exp norway/ or exp sweden/ or exp spain/ or exp switzerland/ or exp australia/ or exp seychelles/ or exp cyprus/ or exp malta/ or exp new zealand/ or exp polynesia/ or exp "antigua and barbuda"/ or exp bahamas/ or exp puerto rico/ or exp "saint kitts and nevis"/ or exp "trinidad and tobago"/ or exp united states virgin islands/ or exp British Virgin Islands/ or new caledonia/ or exp guam/ or exp palau/

20 (Andorra* or "Antiqua and Barbuda" or Antigua* or Barbuda* or Aruba* or Australia* or Austria* or Baham* or Bahrain* or Belgium or Belgian* or Bermud* or "British Virgin Island*" or "Brunei Darussalam" or Bruneian* or Canad* or "Cayman Island*" or Caymanian* or "Channel Island*" or Chile* or Croatia* or Curacao* or Cyprus or Cypriot* or Czech* or "Czech Republic*" or Denmark or Danish or Dane or Estoni* or "Faroe Island*" or Faroese or Finland* or Finnish or Fin* or France or French or "French Polynesia*" or German* or Gibraltar* or Greece or Greek* or Greenland* or Guam* or "Hong Kong" or Hongkonger* or "Hong Konger" or "Hong Kongese" or Hungar* or Iceland* or Ireland or Irish or "Isle of Man" or Manx* or Israel* or Ital* or Japan* or Korea* or Kuwait* or Lativ* or Liechtenstein* or Lithuan* or Luxembourg* or Macao or Macanese or Malta or Maltese or Maltin* or Monaco or Monegasque* or Monacan or Nauru* or Netherland* or Dutch or "New Caledonia*" or "New Zealand*" or "Northern Mariana Island*" or Norway or Norwegian* or Oman* or Palau* or Poland or Polish or Portugal or Portuguese or "Puerto Ric*" or Qatar* or "San Mariano" or Sammarinese or "Saudi Arabia*" or Saudi or Seychell* or Singapore* or "Sint Maarten*" or "Slovak Republic" or Slovak* or Slovenia* or Spain or Spaniard* or "St. Kitts and Nevis" or Kittitian* or Nevisian* or "St Martin*" or "Saint Martin*" or "Saint-Martin*" or Swed* or Switzerland or Swiss or Taiwan* or "Trinidad and Tobago" or Trinid* or Tobago* or "Turks and Caicos Island*" or "United Arab Emirates" or Emirati* or "United Kingdom" or England* or "British" or "United States" or American* or Uruguay* or "Virgin Island*" or "North Americ*" or Europe*).mp. [mp=title, abstract, original title, name of substance word, subject heading word, floating sub-heading word, keyword heading word, organism supplementary concept word, protocol supplementary concept word, rare disease supplementary concept word, unique identifier, synonyms]

21 1 or 2 or 3 or 4

22 5 or 6

23 7 or 8 or 9 or 10 or 11 or 12 or 13 or 14 or 15 or 16

24 17 or 18 or 19 or 20

25 21 and 22 and 23 and 24

26 limit 25 to (english language and yr="2000 -Current")

**Embase Search Strategy**

1 exp infant/ or exp "minor (person)"/ or exp pediatrics/ or exp juvenile/

2 (child* or adolesc* or teen* or preteen* or pre-teen* or tween* or juvenile* or youth* or minor* or kid* or "school age" or schoolchild* or "school child*" or toddler* or infan* or neonat* or baby or babies or newborn* or "new born*" or p?ediatric* or prepubesc* or pre-pubesc* or pubescen*).ti,ab.

3 exp caregiver/ or exp grandparent/ or exp parent/ or exp legal guardian/ or exp sibling/

4 (caregiver* or "care giver" or carer* or caretaker* or "care taker*" or guardian* or parent* or stepparent or mom* or mother* or father* or Dad* or stepfather* or stepmother* or grandparent* or grandm* or grandpa* or grandfather* or sibling* or sister* or brother*).ti,ab.

5 congenital heart malformation/ or congenital heart disease/ or exp aortic coarctation/ or exp aorto-ventricular tunnel/ or exp heart right ventricle dysplasia/ or exp bicuspid aortic valve/ or exp cor triatriatum/ or exp coronary artery anomaly/ or exp dextrocardia/ or exp patent ductus arteriosus/ or exp Ebstein anomaly/ or exp ectopia cordis/ or exp Eisenmenger complex/ or exp heart septum defect/ or exp hypoplastic left heart syndrome/ or exp ventricular noncompaction/ or exp quadricuspid aortic valve/ or exp Fallot tetralogy/ or exp great vessels transposition/ or exp tricuspid valve atresia/ or exp heart atrium septum defect/ or exp heart single ventricle/ or exp persistent left superior vena cava/ or exp pulmonary valve atresia/ or exp vascular ring/ or exp scimitar syndrome/ or exp congestive cardiomyopathy/ or exp hypertrophic cardiomyopathy/ or exp restrictive cardiomyopathy/ or exp aortic valve disease/ or exp heart valve prolapse/ or exp tricuspid valve disease/ or exp mitral valve disease/ or exp pulmonary valve disease/ or exp arterial trunk/ or exp Fontan procedure/

6 ("congenital heart disease" or "congenital heart defect*" or "cyanotic heart disease" or "cyanotic heart defect*" or "p?ediatric heart disease" or "aortic valve stenosis" or "aortic atresia" or "quadricuspid aortic valve" or "bicuspid aortic valve" or "atrial septal defect*" or "aortic coarctation" or "coarctation of the aorta" or "hypoplastic aortic arch" or "interrupted aortic arch" or "atrioventricular canal" or "atrioventricular tunnel" or "atrioventricular septal defect" or "dextro transposition of the great arteries" or "levo transposition of the great arteries" or "transposition of the great arteries" or "transposition of the great vessels" or "Ebstein* anomaly" or "patent ductus arteriosus" or "patent foramen ovale" or "pulmonary valve stenosis" or "Tetralogy of Fallot" or "Trilogy of Fallot" or "Truncus Arteriosus" or hemitruncus or "single ventricle" or "univentricular heart" or "anomalous pulmonary venous" or "Scimitar syndrome" or "pulmonary venous stenosis" or "ventricular septal defect*" or "hypoplastic left heart syndrome" or "pulmonary atresia" or "pulmonary stenosis" or "tricuspid atresia" or "tricuspid stenosis" or "double outlet right ventricle" or "double inlet left ventricle" or "mitral atresia" or "mitral stenosis" or "congenital cardiomyopathy" or "p?ediatric cardiomyopathy" or "dilated cardiomyopathy" or "restricted cardiomyopathy" or " hypertrophic cardiomyopathy" or "arrhythmogenic right ventricular dysplasia" or "mitochondrial noncompaction" or "mitral valve disease" or "absent pulmonary valve" or "hypoplastic right heart syndrome" or Fontan or "vascular ring" or "Cor Triatriatum" or "pulmonary artery stenosis" or "pulmonary artery sling" or "double aortic arch" or "absent heart valve" or "Shone* complex*" or "aortic insufficiency" or "tricuspid insufficiency" or "mitral insufficiency" or "pulmonary insufficiency" or "pulmonary regurgitation" or "tricuspid regurgitation" or "mitral regurgitation" or "aortic regurgitation" or dextrocardia or "crisscross heart" or "Eisenmenger complex" or "Eisenmenger Syndrome" or "Ectopia Cordis" or "atrial isomerism*").ti,ab.

7 exp psychosocial intervention/ or exp social psychology/ or exp psychosocial care/

8 (psychosocial or psycho-social).ti,ab.

9 exp psychological adjustment/ or attitude/ or exp attitude to death/ or exp attitude to health/ or exp catastrophizing/ or exp optimism/ or exp pessimism/ or exp respect/ or exp stereotyping/ or exp humor/ or exp behavior/ or exp child rearing/ or exp defense mechanism/ or exp emotion/ or exp human development/ or exp mental capacity/ or exp motivation/ or exp behavior disorder/ or exp personality/ or exp alcohol abstinence/ or exp "psychological and psychiatric procedures"/ or exp mental disease/ or exp endurance/ or exp exercise/ or exp fitness/ or exp fatigue/ or exp alcoholism/ or exp substance abuse/

10 ("lived experience*" or "live through" or attitude* or psych* or mental* or "mental process*" or "mental health" or "mental disorder*" or mind* or feel* or thought* or emotion* or "social cognition*" or "cognitive process*" or depression or behavio?r* or anxiety or anxious or "attention deficit" or alcoholi* or lifestyle or suicid* or "cognitive deficit*" or "self-esteem" or "self-concept" or "body image" or "body perception" or fear* or "stress disorder" or anger or distrust or "medical trauma" or "physical trauma" or addict* or panic or "sexual activit*" or "sex life" or "sex drive" or "drug use" or "drug abuse*" or grief or griev* or bereave* or wellness or wellbeing or well-being or "physical health" or "physical shape" or "physical condition*" or "physical fitness*" or endurance or fatigue or "exercise tolerance" or "coping skill*" or "coping strateg*").ti,ab.

11 exp social justice/ or exp public opinion/ or exp childhood adversity/ or exp bullying/ or exp violence/ or social problem/ or exp divorce/ or exp emotional abuse/ or exp poverty/ or exp social segregation/ or exp suicide/ or exp underage drinking/ or exp social structure/ or exp social welfare/ or exp cultural anthropology/ or exp ethnic group/ or exp family relation/ or exp family separation/ or exp social hierarchy/ or exp medicalization/ or exp minority group/ or exp secularism/ or exp social capital/ or exp social status/ or exp social environment/ or exp social isolation/ or exp social exclusion/ or exp social norm/ or exp vulnerable population/ or exp Social Vulnerability Index/ or exp socioeconomics/ or exp social status/ or exp needs assessment/ or exp return to school/ or exp transcultural care/ or exp integrated health care system/ or exp health care access/ or exp health care disparity/ or exp health disparity/ or exp educational status/

12 (friendship* or "family relation*" or "personal relationship*" or "interpersonal relationship*" or "social environment*" or "social factor*" or "social barrier*" or "social inclusion" or "social network*" or employer* or "social group*" or "social relationship*" or "professional relationship*" or "intimate relationship*" or "sexual partner" or "marital status" or "social structure*" or "society as a whole" or "socioeconomic status" or "political system*" or "judicial system*" or "legal system*" or "health* system*" or "health care system*" or "healthcare system*" or "health* program*" or "health care program*" or "healthcare group*" or "health care group*" or "education system*" or "school system" or "cultural environment*" or patriarchy or marginali* or discriminat* or judgement or "social isolation" or prejudice* or bias* or bigotry or intolerance* or "narrow mindedness" or inequalit* or inequit* or disadvantage* or disempower* or empower* or stigma* or vulnerable or bullying or bullied or oppress* or persecute* or "traumatic event*" or workplace* or "work place* or social justice" or "social injustice*").ti,ab.

13 employment/ or exp supported employment/ or exp unemployment/ or exp workplace/ or exp income/

14 (finance* or income or "finance* service*" or "employ* service*" or "finance* resource* " or "employ* resource*" or "material resource*" or employ* or unemploy* or job? or "distribution of material resource*" or "social* disadvantage*" or "neo material" or money or "pecuniary fund*" or bankrupt* or welfare or "socioeconomic factor*").ti,ab.

15 exp religion/

16 (spiritual* or religion* or religious* or Ignatian or otherworldliness or unearthliness or incorporeality or piousness or devoutness or holiness or religiosity or pietism or piety or reverence or transcendence or "relationship to the transcendent" or "religious affiliation*").ti,ab.

17 exp developed country/

18 ("high income countr*" or "high-income countr*" or "high income nation*" or "high-income nation*" or "developed countr*" or "developed nation*" or "first world countr*" or "first world nation*").mp. [mp=title, abstract, heading word, drug trade name, original title, device manufacturer, drug manufacturer, device trade name, keyword heading word, floating subheading word, candidate term word]

19 exp Aruba/ or exp Curacao/ or North America/ or exp Canada/ or exp Greenland/ or exp United States/ or exp Chile/ or exp Uruguay/ or exp Brunei Darussalam/ or exp Singapore/ or exp Bahrain/ or exp Israel/ or exp Kuwait/ or exp Oman/ or exp Qatar/ or exp Saudi Arabia/ or exp United Arab Emirates/ or exp Hong Kong/ or exp Macao/ or exp Japan/ or exp Korea/ or exp Taiwan/ or Europe/ or exp Andorra/ or exp Austria/ or exp Belgium/ or exp Estonia/ or exp Latvia/ or exp Lithuania/ or exp Croatia/ or exp Czech Republic/ or exp Hungary/ or exp Poland/ or exp Slovakia/ or exp Slovenia/ or exp France/ or exp Germany/ or exp Gibraltar/ or exp United Kingdom/ or exp Channel Islands/ or exp England/ or exp Northern Ireland/ or exp Greece/ or exp Ireland/ or exp Italy/ or exp Liechtenstein/ or exp Luxembourg/ or exp Monaco/ or exp Netherlands/ or exp Portugal/ or exp San Marino/ or exp Denmark/ or exp Finland/ or exp Iceland/ or exp Norway/ or exp Sweden/ or exp Spain/ or exp Switzerland/ or exp Australia/ or exp Seychelles/ or exp Cyprus/ or exp Malta/ or exp New Zealand/ or exp French Polynesia/ or exp "Antigua and Barbuda"/ or exp Bahamas/ or exp Puerto Rico/ or exp "Saint Kitts and Nevis"/ or exp "Trinidad and Tobago"/ or exp "Virgin Islands (U.S.)"/ or exp "Virgin Islands (British)"/ or exp New Caledonia/ or exp Guam/ or exp Palau/ or exp "Saint Martin (Dutch)"/

20 (Andorra* or "Antiqua and Barbuda" or Antigua* or Barbuda* or Aruba* or Australia* or Austria* or Baham* or Bahrain* or Belgium or Belgian* or Bermud* or "British Virgin Island*" or "Brunei Darussalam" or Bruneian* or Canad* or "Cayman Island*" or Caymanian* or "Channel Island*" or Chile* or Croatia* or Curacao* or Cyprus or Cypriot* or Czech* or "Czech Republic*" or Denmark or Danish or Dane or Estoni* or "Faroe Island*" or Faroese or Finland* or Finnish or Fin* or France or French or "French Polynesia*" or German* or Gibraltar* or Greece or Greek* or Greenland* or Guam* or "Hong Kong" or Hongkonger* or "Hong Konger" or "Hong Kongese" or Hungar* or Iceland* or Ireland or Irish or "Isle of Man" or Manx* or Israel* or Ital* or Japan* or Korea* or Kuwait* or Lativ* or Liechtenstein* or Lithuan* or Luxembourg* or Macao or Macanese or Malta or Maltese or Maltin* or Monaco or Monegasque* or Monacan or Nauru* or Netherland* or Dutch or "New Caledonia*" or "New Zealand*" or "Northern Mariana Island*" or Norway or Norwegian* or Oman* or Palau* or Poland or Polish or Portugal or Portuguese or "Puerto Ric*" or Qatar* or "San Mariano" or Sammarinese or "Saudi Arabia*" or Saudi or Seychell* or Singapore* or "Sint Maarten*" or "Slovak Republic" or Slovak* or Slovenia* or Spain or Spaniard* or "St. Kitts and Nevis" or Kittitian* or Nevisian* or "St Martin*" or "Saint Martin*" or "Saint-Martin*" or Swed* or Switzerland or Swiss or Taiwan* or "Trinidad and Tobago" or Trinid* or Tobago* or "Turks and Caicos Island*" or "United Arab Emirates" or Emirati* or "United Kingdom" or England* or "British" or "United States" or American* or Uruguay* or "Virgin Island*" or "North Americ*" or Europe*).mp. [mp=title, abstract, heading word, drug trade name, original title, device manufacturer, drug manufacturer, device trade name, keyword heading word, floating subheading word, candidate term word]

21 1 or 2 or 3 or 4

22 5 or 6

23 7 or 8 or 9 or 10 or 11 or 12 or 13 or 14 or 15 or 16

24 17 or 18 or 19 or 20

25 21 and 22 and 23 and 24

26 limit 25 to (books or chapter or conference abstract or conference paper or "conference review" or editorial or erratum or letter or note)

27 25 not 26

28 limit 27 to (english language and yr="2000 -Current")

**APA PsycInfo Search Strategy**

1 exp childhood development/ or exp adolescent development/ or exp Infant Development/ or exp pediatrics/

2 (child* or adolesc* or teen* or preteen* or pre-teen* or tween* or juvenile* or youth* or minor* or kid* or "school age" or schoolchild* or "school child*" or toddler* or infan* or neonat* or baby or babies or newborn* or "new born*" or p?ediatric* or prepubesc* or pre-pubesc* or pubescen*).ti,ab.

3 exp caregivers/ or exp grandparents/ or exp Parents/ or exp guardianship/ or exp Siblings/

4 (caregiver* or "care giver" or carer* or caretaker* or "care taker*" or guardian* or parent* or stepparent or mom* or mother* or father* or Dad* or stepfather* or stepmother* or grandparent* or grandm* or grandpa* or grandfather* or sibling* or sister* or brother*).ti,ab.

5 ("congenital heart disease" or "congenital heart defect*" or "cyanotic heart disease" or "cyanotic heart defect*" or "p?ediatric heart disease" or "aortic valve stenosis" or "aortic atresia" or "quadricuspid aortic valve" or "bicuspid aortic valve" or "atrial septal defect*" or "aortic coarctation" or "coarctation of the aorta" or "hypoplastic aortic arch" or "interrupted aortic arch" or "atrioventricular canal" or "atrioventricular tunnel" or "atrioventricular septal defect" or "dextro transposition of the great arteries" or "levo transposition of the great arteries" or "transposition of the great arteries" or "transposition of the great vessels" or "Ebstein* anomaly" or "patent ductus arteriosus" or "patent foramen ovale" or "pulmonary valve stenosis" or "Tetralogy of Fallot" or "Trilogy of Fallot" or "Truncus Arteriosus" or hemitruncus or "single ventricle" or "univentricular heart" or "anomalous pulmonary venous" or "Scimitar syndrome" or "pulmonary venous stenosis" or "ventricular septal defect*" or "hypoplastic left heart syndrome" or "pulmonary atresia" or "pulmonary stenosis" or "tricuspid atresia" or "tricuspid stenosis" or "double outlet right ventricle" or "double inlet left ventricle" or "mitral atresia" or "mitral stenosis" or "congenital cardiomyopathy" or "p?ediatric cardiomyopathy" or "dilated cardiomyopathy" or "restricted cardiomyopathy" or " hypertrophic cardiomyopathy" or "arrhythmogenic right ventricular dysplasia" or "mitochondrial noncompaction" or "mitral valve disease" or "absent pulmonary valve" or "hypoplastic right heart syndrome" or Fontan or "vascular ring" or "Cor Triatriatum" or "pulmonary artery stenosis" or "pulmonary artery sling" or "double aortic arch" or "absent heart valve" or "Shone* complex*" or "aortic insufficiency" or "tricuspid insufficiency" or "mitral insufficiency" or "pulmonary insufficiency" or "pulmonary regurgitation" or "tricuspid regurgitation" or "mitral regurgitation" or "aortic regurgitation" or dextrocardia or "crisscross heart" or "Eisenmenger complex" or "Eisenmenger Syndrome" or "Ectopia Cordis" or "atrial isomerism*").ti,ab.

6 exp Psychosocial Development/ or exp Psychosocial Readjustment/ or exp Psychosocial Assessment/ or exp Psychosocial Outcomes/ or exp Psychosocial Factors/ or exp psychosocial rehabilitation/ or Psychosocial Factors/ or exp Social Functioning/ or exp social support/ 243457

7 (psychosocial or psycho-social).ti,ab.

8 exp emotional adjustment/ or attitudes/ or exp Death Attitudes/ or exp health attitudes/ or exp catastrophizing/ or exp Pessimism/ or exp Optimism/ or exp respect/ or exp Stereotyped Attitudes/ or exp Humor/ or exp Behavior/ or exp childrearing practices/ or exp Defense Mechanisms/ or exp Emotions/ or exp human development/ or exp cognitive processes/ or exp Motivation/ or exp cognitive impairment/ or exp Personality/ or exp Social Psychology/ or exp Drug Abuse/ or exp Alcohol Abuse/ or exp Alcoholism/ or exp Mental Disorders/ or exp Physical Endurance/ or exp Physical Activity/ or exp Exercise/ or exp Fatigue/

9 ("lived experience*" or "live through" or attitude* or psych* or mental* or "mental process*" or "mental health" or "mental disorder*" or mind* or feel* or thought* or emotion* or "social cognition*" or "cognitive process*" or depression or behavio?r* or anxiety or anxious or "attention deficit" or alcoholi* or lifestyle or suicid* or "cognitive deficit*" or "self-esteem" or "self-concept" or "body image" or "body perception" or fear* or "stress disorder" or anger or distrust or "medical trauma" or "physical trauma" or addict* or panic or "sexual activit*" or "sex life" or "sex drive" or "drug use" or "drug abuse*" or grief or griev* or bereave* or wellness or wellbeing or well-being or "physical health" or "physical shape" or "physical condition*" or "physical fitness*" or endurance or fatigue or "exercise tolerance" or "coping skill*" or "coping strateg*").ti,ab.

10 exp Social Justice/ or exp Public Opinion/ or social issues/ or exp childhood adversity/ or exp Bullying/ or exp dangerousness/ or exp Divorce/ or exp Emotional Abuse/ or exp Poverty/ or exp social integration/ or exp Suicide/ or exp Underage Drinking/ or exp Social Structure/ or exp "welfare services (government)"/ or exp Community Services/ or exp "Culture (Anthropological)"/ or exp Sociocultural Factors/ or exp "Racial and Ethnic Groups"/ or exp Minority Groups/ or exp Socioeconomic Status/ or exp "Racial and Ethnic Differences"/ or exp Health Disparities/ or exp Family Relations/ or exp Family Separation/ or exp social status/ or exp medical model/ or exp Social Capital/ or exp Social Environments/ or exp Social Isolation/ or exp marginalization/ or exp Social Norms/ or exp at risk populations/ or exp Sociocultural Factors/ or exp Socioeconomic Factors/ or exp Needs Assessment/ or exp Reentry Students/ or exp cross cultural treatment/ or exp Cultural Sensitivity/ or exp integrated services/ or exp health care access/ or exp Educational Background/ or exp Educational Attainment Level/ or exp Academic Achievement/

11 (friendship* or "family relation*" or "personal relationship*" or "interpersonal relationship*" or "social environment*" or "social factor*" or "social barrier*" or "social inclusion" or "social network*" or employer* or "social group*" or "social relationship*" or "professional relationship*" or "intimate relationship*" or "sexual partner" or "marital status" or "social structure*" or "society as a whole" or "socioeconomic status" or "political system*" or "judicial system*" or "legal system*" or "health* system*" or "health care system*" or "healthcare system*" or "health* program*" or "health care program*" or "healthcare group*" or "health care group*" or "education system*" or "school system" or "cultural environment*" or patriarchy or marginali* or discriminat* or judgement or "social isolation" or prejudice* or bias* or bigotry or intolerance* or "narrow mindedness" or inequalit* or inequit* or disadvantage* or disempower* or empower* or stigma* or vulnerable or bullying or bullied or oppress* or persecute* or "traumatic event*" or workplace* or "work place* or social justice" or "social injustice*").ti,ab.

12 exp employment status/ or exp Supported Employment/ or exp Unemployment/ or exp Diversity in the Workplace/ or exp income level/

13 (finance* or income or "finance* service*" or "employ* service*" or "finance* resource* " or "employ* resource*" or "material resource*" or employ* or unemploy* or job? or "distribution of material resource*" or "social* disadvantage*" or "neo material" or money or "pecuniary fund*" or bankrupt* or welfare or "socioeconomic factor*").ti,ab.

14 exp Religiosity/ or exp Well Being/ or exp Religion/ or exp Religious Beliefs/ or exp Spirituality/

15 (spiritual* or religion* or religious* or Ignatian or otherworldliness or unearthliness or incorporeality or piousness or devoutness or holiness or religiosity or pietism or piety or reverence or transcendence or "relationship to the transcendent" or "religious affiliation*").ti,ab.

16 exp Developed Countries/

17 ("high income countr*" or "high-income countr*" or "high income nation*" or "high-income nation*" or "developed countr*" or "developed nation*" or "first world countr*" or "first world nation*").mp. [mp=title, abstract, heading word, table of contents, key concepts, original title, tests & measures, mesh word]

18 (Andorra* or "Antiqua and Barbuda" or Antigua* or Barbuda* or Aruba* or Australia* or Austria* or Baham* or Bahrain* or Belgium or Belgian* or Bermud* or "British Virgin Island*" or "Brunei Darussalam" or Bruneian* or Canad* or "Cayman Island*" or Caymanian* or "Channel Island*" or Chile* or Croatia* or Curacao* or Cyprus or Cypriot* or Czech* or "Czech Republic*" or Denmark or Danish or Dane or Estoni* or "Faroe Island*" or Faroese or Finland* or Finnish or Fin* or France or French or "French Polynesia*" or German* or Gibraltar* or Greece or Greek* or Greenland* or Guam* or "Hong Kong" or Hongkonger* or "Hong Konger" or "Hong Kongese" or Hungar* or Iceland* or Ireland or Irish or "Isle of Man" or Manx* or Israel* or Ital* or Japan* or Korea* or Kuwait* or Lativ* or Liechtenstein* or Lithuan* or Luxembourg* or Macao or Macanese or Malta or Maltese or Maltin* or Monaco or Monegasque* or Monacan or Nauru* or Netherland* or Dutch or "New Caledonia*" or "New Zealand*" or "Northern Mariana Island*" or Norway or Norwegian* or Oman* or Palau* or Poland or Polish or Portugal or Portuguese or "Puerto Ric*" or Qatar* or "San Mariano" or Sammarinese or "Saudi Arabia*" or Saudi or Seychell* or Singapore* or "Sint Maarten*" or "Slovak Republic" or Slovak* or Slovenia* or Spain or Spaniard* or "St. Kitts and Nevis" or Kittitian* or Nevisian* or "St Martin*" or "Saint Martin*" or "Saint-Martin*" or Swed* or Switzerland or Swiss or Taiwan* or "Trinidad and Tobago" or Trinid* or Tobago* or "Turks and Caicos Island*" or "United Arab Emirates" or Emirati* or "United Kingdom" or England* or "British" or "United States" or American* or Uruguay* or "Virgin Island*" or "North Americ*" or Europe*).mp. [mp=title, abstract, heading word, table of contents, key concepts, original title, tests & measures, mesh word]

19 1 or 2 or 3 or 4

20 6 or 7 or 8 or 9 or 10 or 11 or 12 or 13 or 14 or 15

21 16 or 17 or 18

22 5 and 19 and 20 and 21

23 limit 22 to (english language and yr="2000 -Current")

Note: CHD not available as Mesh closest congenital disorders- not used as too broad

Individual countries also not available as MeSH

**CINAHL Search Strategy**

| **#** | **Query** | **Limiters/Expanders** |
| --- | --- | --- |
| S1 | (MH "Adolescence+") OR (MH "Child+") OR (MH "Infant+") OR (MH "Minors (Legal)") OR (MH "Pediatrics+") | Search modes - Find all my search terms |
| S2 | child* OR adolesc* OR teen* OR preteen* OR pre-teen* OR tween* OR juvenile* OR youth* OR minor* OR kid* OR "school age" OR schoolchild* OR "school child*" OR toddler* OR infan* OR neonat* OR baby OR babies OR newborn* OR "new born*" OR p#ediatric* OR prepubesc* OR pre-pubesc* OR pubescen* | Search modes - Find all my search terms |
| S3 | (MH "Caregivers") OR (MH "Grandparents") OR (MH "Parents+") OR (MH "Guardianship, Legal+") OR (MH "Siblings") | Search modes - Find all my search terms |
| S4 | caregiver* OR "care giver" OR carer* OR caretaker* OR "care taker*" OR guardian* OR parent* OR stepparent OR mom* OR mother* OR father* OR Dad* OR stepfather* OR stepmother* OR grandparent* OR grandm* OR grandpa* OR grandfather* OR sibling* OR sister* OR brother* | Search modes - Find all my search terms |
| S5 | (MH "Heart Defects, Congenital") OR (MH "Aortic Coarctation") OR (MH "Aortic Valve Stenosis+") OR (MH "Aortic Valve Insufficiency") OR (MH "Aortic Valve Diseases+") OR (MH "Quadricuspid Aortic Valve") OR (MH "Heart Septal Defects+") OR (MH "Heart Septal Defects, Ventricular") OR (MH "Heart Septal Defects, Atrial") OR (MH "Aortopulmonary Septal Defect") OR (MH "Endocardial Cushion Defects") OR (MH "Arrhythmogenic Right Ventricular Dysplasia") OR (MH "Bicuspid Aortic Valve Disease") Or (MH "Bland-White-Garland Syndrome") OR (MH "Dextrocardia") OR (MH "Ductus Arteriosus, Patent") OR (MH "Ebstein's Anomaly") OR (MH "Ectopia Cordis") OR (MH "Hypoplastic Left Heart Syndrome") OR (MH "Tetralogy of Fallot") OR (MH "Transposition of Great Arteries+") OR (MH "Congenitally Corrected Transposition of the Great Arteries") OR (MH "Tricuspid Atresia") OR (MH "Pulmonary Atresia") OR (MH "Cor Triatriatum") OR (MH "Univentricular Heart") OR (MH "Persistent Left Superior Vena Cava") OR (MH "Scimitar Syndrome") OR (MH "Cardiomyopathy, Dilated") OR (MH "Cardiomyopathy, Hypertrophic") OR (MH "Mitral Valve Prolapse") OR (MH "Mitral Valve Stenosis") OR (MH "Mitral Valve Insufficiency") OR (MH "Pulmonary Valve Stenosis+") OR (MH "Pulmonary Valve Diseases+") OR (MH "Tricuspid Valve Diseases") OR (MH "Truncus Arteriosus, Persistent") | Search modes - Find all my search terms |
| S6 | \| "congenital heart disease" OR "congenital heart defect*" OR "cyanotic heart disease" OR "cyanotic heart defect*" OR "p#ediatric heart disease" OR "aortic valve stenosis" OR "aortic atresia" OR "quadricuspid aortic valve" OR "bicuspid aortic valve" OR "atrial septal defect*" OR "aortic coarctation" OR "coarctation of the aorta" OR "hypoplastic aortic arch" OR "interrupted aortic arch" OR "atrioventricular canal" OR "atrioventricular tunnel" OR "atrioventricular septal defect" OR "dextro transposition of the great arteries" OR "levo transposition of the great arteries" OR "transposition of the great arteries" OR "transposition of the great vessels" OR "Ebstein* anomaly" OR "patent ductus arteriosus" OR "patent foramen ovale" OR "pulmonary valve stenosis" OR "Tetralogy of Fallot" OR "Trilogy of Fallot" OR "Truncus Arteriosus" OR hemitruncus OR "single ventricle" OR "univentricular heart" OR "anomalous pulmonary venous" OR "Scimitar syndrome" OR "pulmonary venous stenosis" OR "ventricular septal defect*" OR "hypoplastic left heart syndrome" OR "pulmonary atresia" OR "pulmonary stenosis" OR "tricuspid atresia" OR "tricuspid stenosis" OR "double outlet right ventricle" OR "double inlet left ventricle" OR "mitral atresia" OR "mitral stenosis" OR "congenital cardiomyopathy" OR "p#ediatric cardiomyopathy" OR "dilated cardiomyopathy" OR "restricted cardiomyopathy" OR " hypertrophic cardiomyopathy" OR "arrhythmogenic right ventricular dysplasia" OR "mitochondrial noncompaction" OR "mitral valve disease" OR "absent pulmonary valve" OR "hypoplastic right heart syndrome" OR Fontan OR "vascular ring" OR "Cor Triatriatum" OR "pulmonary artery stenosis" OR "pulmonary artery sling" OR "double aortic arch" OR "absent heart valve" OR "Shone* complex*" OR "aortic insufficiency" OR "tricuspid insufficiency" OR "mitral insufficiency" OR "pulmonary insufficiency" OR "pulmonary regurgitation" OR "tricuspid regurgitation" OR "mitral regurgitation" OR "aortic regurgitation" OR dextrocardia OR "crisscross heart" OR "Eisenmenger complex" OR "Eisenmenger Syndrome" OR "Ectopia Cordis" OR "atrial isomerism*" \| Search modes - Find all my search terms \| Interface - EBSCOhost Research Databases Search Screen - Advanced Search Database - CINAHL Plus with Full Text \| 44,670 \| \| --- \| --- \| --- \| --- \| |  |
| S7 | (MH "Support, Psychosocial+") OR (MH "Psychosocial Intervention") OR (MH "Psychosocial Functioning") OR (MH "Psychosocial Adjustment to Illness Scale") OR (MH "Psychosocial Aspects of Illness+") | Search modes - Find all my search terms |
| S8 | psychosocial OR psycho-social | Search modes - Find all my search terms |
| S9 | (MH "Adaptation, Psychological+") OR (MH "Attitude") OR (MH "Attitude to Illness+") OR (MH "Attitude to Health+") OR (MH "Attitude to Death+") OR (MH "Catastrophization") OR (MH "Optimism") OR (MH "Pessimism") OR (MH "Respect") OR (MH "Stereotyping") OR (MH "Stigma") OR (MH "Wit and Humor") OR (MH "Behavior+") OR (MH "Child Rearing+") OR (MH "Defense Mechanisms+") OR (MH "Emotions+")OR (MH "Human Development+") OR MH "Motivation+") OR (MH "Neurobehavioral Manifestations+") OR (MH "Personality+") OR (MH "Psychology, Social+") OR (MH "Mental Disorders+") OR (MH "Physical Endurance+") OR (MH "Exertion+") OR (MH "Physical Fitness+") OR (MH "Fatigue+") OR (MH "Alcoholism") OR (MH "Substance Abuse, Intravenous") OR (MH "Substance Abuse+") | Search modes - Find all my search terms |
| S10 | "lived experience*" OR "live through" OR attitude* OR psych* OR mental* OR "mental process*" OR "mental health" OR "mental disorder*" OR mind* OR feel* OR thought* OR emotion* OR "social cognition*" OR "cognitive process*" OR depression OR behavio#r* OR anxiety OR anxious OR "attention deficit" OR alcoholi* OR lifestyle OR suicid* OR "cognitive deficit*" OR "self-esteem" OR "self-concept" OR "body image" OR "body perception" OR fear* OR "stress disorder" OR anger OR distrust OR "medical trauma" OR "physical trauma" OR addict* OR panic OR "sexual activit*" OR "sex life" OR "sex drive" OR "drug use" OR "drug abuse*" OR grief OR griev* OR bereave* OR wellness OR wellbeing OR well-being OR "physical health" OR "physical shape" OR "physical condition*" OR "physical fitness*" OR endurance OR fatigue OR "exercise tolerance" OR "coping skill*" OR "coping strateg*" | Search modes - Find all my search terms |
| S11 | (MH "Social Justice") OR (MH "Public Opinion") OR (MH "Social Problems") OR (MH "Adverse Childhood Experiences") OR (MH "Bullying+") OR (MH "Risk Taking Behavior+") OR (MH "Divorce") OR (MH "Emotional Abuse") OR (MH "Poverty+") OR (MH "Suicide+") OR (MH "Social Welfare+") OR (MH "Health Resource Allocation") OR (MH "Culture+") OR (MH "Racial Equality") OR (MH "Ethnic Groups+") OR (MH "Family Relations+") OR (MH "Family Separation") OR (MH "Minority Groups")OR (MH "Humanism") OR (MH "Social Capital") OR (MH "Social Environment+") OR (MH "Social Isolation+") OR (MH "Social Norms") OR (MH "Socioeconomic Factors+") OR (MH "Needs Assessment") OR (MH "School Re-Entry") OR (MH "Cultural Competence") OR (MH "Health Care Delivery, Integrated") OR (MH "Health Services Accessibility+") OR (MH "Healthcare Disparities")OR (MH "Educational Status") | Search modes - Find all my search terms |
| S12 | friendship* OR "family relation*" OR "personal relationship*" OR "interpersonal relationship*" OR "social environment*" OR "social factor*" OR "social barrier*" OR "social inclusion" OR "social network*" OR employer* OR "social group*" OR "social relationship*" or "professional relationship*" OR "intimate relationship*" OR "sexual partner" OR "marital status" OR "social structure*" OR "society as a whole" OR "socioeconomic status" OR "political system*" OR "judicial system*" OR "legal system*" OR "health* system*" OR "health care system*" OR "healthcare system*" OR "health* program*" OR "health care program*" OR "healthcare group*" OR "health care group*" OR "education system*" OR "school system" OR "cultural environment*" OR patriarchy OR marginali* OR discriminat* OR judgement OR "social isolation" OR prejudice* OR bias* OR bigotry OR intolerance* OR "narrow mindedness" OR inequalit* OR inequit* OR disadvantage* OR disempower* OR empower* OR stigma* OR vulnerable OR bullying OR bullied OR oppress* OR persecute* OR "traumatic event*" OR workplace* OR "work place*" OR "social justice" OR "social injustice*" | Search modes - Find all my search terms |
| S13 | S13 | (MH "Employment+") OR (MH "Employment, Supported") OR (MH "Unemployment") OR ( MH "Work Environment+") OR (MH "Income+") |
| S14 | finance* OR income OR "finance* service*" OR "employ* service*" OR "finance* resource* " OR "employ* resource*" OR "material resource*" OR employ* OR unemploy* OR job* OR "distribution of material resource*" OR "social* disadvantage*" OR "neo material" OR money OR "pecuniary fund*" OR bankrupt* OR welfare OR "socioeconomic factor*" | Search modes - Find all my search terms |
| S15 | (MH "Religion and Religions+") OR (MH "Religion and Psychology+") | Search modes - Find all my search terms |
| S16 | spiritual* OR religion* OR religious* OR Ignatian OR otherworldliness OR unearthliness OR incorporeality OR piousness OR devoutness OR holiness OR religiosity OR pietism OR piety OR reverence OR transcendence OR "relationship to the transcendent" OR "religious affiliation*" | Search modes - Find all my search terms |
| S17 | (MH "Developed Countries") | Search modes - Find all my search terms |
| S18 | "high income countr*" OR "high-income countr*" OR "high income nation*" OR "high-income nation*" OR "developed countr*" OR "developed nation*" OR "first world countr*" OR "first world nation*" | Search modes - Find all my search terms |
| S19 | (MH "Canada+") OR (MH "North America+")OR (MH "United States+") OR (MH "Chile") OR (MH "Uruguay") OR (MH "Brunei") OR (MH "Singapore")OR (MH "Bahrain") OR (MH "Israel") OR (MH "Kuwait") OR (MH "Oman") OR (MH "Qatar")OR (MH "Saudi Arabia") OR (MH "United Arab Emirates") OR (MH "Hong Kong") OR (MH "Macao") OR (MH "Japan") OR (MH "Korea") OR (MH "Taiwan") OR (MH "Europe") OR (MH "Andorra") OR (MH "Austria") OR (MH "Belgium") OR (MH "Estonia") OR (MH "Latvia")OR (MH "Lithuania") OR (MH "Croatia") OR (MH "Czech Republic") OR (MH "Slovakia") OR (MH "Hungary") OR (MH "Poland") OR (MH "Slovenia") OR (MH "France") OR (MH "Germany+") OR (MH "Gibraltar") OR (MH "United Kingdom+") OR (MH "England") OR (MH "Northern Ireland") OR (MH "Ireland") OR (MH "Greece")OR (MH "Italy") OR (MH "Liechtenstein")OR (MH "Luxembourg") OR (MH "Monaco") OR (MH "Portugal") OR (MH "Netherlands") OR (MH "San Marino") OR (MH "Greenland") OR (MH "Finland")OR (MH "Iceland") Or (MH "Norway") OR (MH "Sweden")OR (MH "Spain") OR (MH "Switzerland") OR (MH "Australia+")OR (MH "Mediterranean Islands") OR (MH "Indian Ocean Islands+") OR (MH "New Zealand") OR (MH "Polynesia+") OR (MH "Antigua") OR (MH "Bahamas") OR (MH "Puerto Rico") OR (MH "Trinidad and Tobago") OR (MH "Virgin Islands of the United States") OR (MH "West Indies+") OR (MH "Melanesia+") OR (MH "Guam") | Search modes - Find all my search terms |
| S20 | Andorra* OR "Antiqua and Barbuda" OR Antigua* OR Barbuda* OR Aruba* OR Australia* OR Austria* OR Baham* OR Bahrain* OR Belgium OR Belgian* OR Bermud* OR "British Virgin Island*" OR "Brunei Darussalam" OR Bruneian* OR Canad* OR "Cayman Island*" OR Caymanian* OR "Channel Island*" OR Chile* OR Croatia* OR Curacao* OR Cyprus OR Cypriot* OR Czech* OR "Czech Republic*" OR Denmark OR Danish OR Dane OR Estoni* OR "Faroe Island*" OR Faroese OR Finland* OR Finnish OR Fin* OR France OR French OR "French Polynesia*" OR German* OR Gibraltar* OR Greece OR Greek* OR Greenland* OR Guam* OR "Hong Kong" OR Hongkonger* OR "Hong Konger" OR "Hong Kongese" OR Hungar* OR Iceland* OR Ireland OR Irish OR "Isle of Man" OR Manx* OR Israel* OR Ital* OR Japan* OR Korea* OR Kuwait* OR Lativ* OR Liechtenstein* OR Lithuan* OR Luxembourg* OR Macao OR Macanese OR Malta OR Maltese OR Maltin* OR Monaco OR Monegasque* OR Monacan OR Nauru* OR Netherland* OR Dutch OR "New Caledonia*" OR "New Zealand*" OR "Northern Mariana Island*" OR Norway OR Norwegian* OR Oman* OR Palau* OR Poland OR Polish OR Portugal OR Portuguese OR "Puerto Ric*" OR Qatar* OR "San Mariano" OR Sammarinese OR "Saudi Arabia*" OR Saudi OR Seychell* OR Singapore* OR "Sint Maarten*" OR "Slovak Republic" OR Slovak* OR Slovenia* OR Spain OR Spaniard* OR "St. Kitts and Nevis" OR Kittitian* OR Nevisian* OR "St Martin*" OR "Saint Martin*" Or "Saint-Martin*" OR Swed* OR Switzerland OR Swiss OR Taiwan* OR "Trinidad and Tobago" OR Trinid* OR Tobago* OR "Turks and Caicos Island*" OR "United Arab Emirates" OR Emirati* OR "United Kingdom" OR England* OR "British" OR "United States" OR American* OR Uruguay* OR "Virgin Island*" OR "North Americ*" OR Europe* | Search modes - Find all my search terms |
| S21 | S1 OR S2 OR S3 OR S4 | Search modes - Find all my search terms |
| S22 | S5 OR S6 | Search modes - Find all my search terms |
| S23 | S7 OR S8 OR S9 OR S10 OR S11 OR S12 OR S13 OR S14 OR S15 OR S16 | Search modes - Find all my search terms |
| S24 | S17 OR S18 OR S19 OR S20 | Search modes - Find all my search terms |
| S25 | S21 AND S22 AND S23 AND S24 | Search modes - Find all my search terms |
| S26 | S21 AND S22 AND S23 AND S24 | Limiters - Scholarly (Peer Reviewed) Journals; English Language; Published Date: 20000101- Search modes - Find all my search terms |

**CENTRAL Search Strategy**

ID Search

#1 ((mh "adolescent") or (mh "child") or (mh "infant") or (mh "minor") or (mh "Pediatrics")) (Word variations have been searched)

#2 (child* or adolesc* or teen* or preteen* or pre-teen* or tween* or juvenile* or youth* or minor* or kid* or "school age" or schoolchild* or "school child*" or toddler* or infan* or neonat* or baby or babies or newborn* or "new born*" or p?ediatric* or prepubesc* or pre-pubesc* or pubescen*):ti,ab,kw

#3 ((mh "caregivers") or (mh "grandparents") or (mh "parents") or (mh "Legal Guardians") or (mh "Siblings"))

#4 (caregiver* or "care giver" or carer* or caretaker* or "care taker*" or guardian* or parent* or stepparent or mom* or mother* or father* or Dad* or stepfather* or stepmother* or grandparent* or grandm* or grandpa* or grandfather* or sibling* or sister* or brother*):ti,ab,kw

#5 (mh "heart defects, congenital") or (mh "aortic coarctation") or (mh "aortico-ventricular tunnel") or (mh "arrhythmogenic right ventricular dysplasia") or (mh "bicuspid aortic valve disease") or (mh "cor triatriatum") or (mh "anomalous left coronary artery") or (mh "dextrocardia") or (mh "ductus arteriosus, patent") or (mh "ebstein anomaly") or (mh "ectopia cordis") or (mh "eisenmenger complex") or (mh "heart septal defects") or (mh "hypoplastic left heart syndrome") or (mh "isolated noncompaction of the ventricular myocardium") or (mh "quadricuspid aortic valve") or (mh "tetralogy of fallot") or (mh "transposition of great vessels") or (mh "tricuspid atresia") or (mh "trilogy of fallot") or (mh "univentricular heart") or (mh "persistent left superior vena cava") or (mh "pulmonary atresia") or (mh "scimitar syndrome") or (mh "vascular ring") or (mh "cardiomyopathy, dilated") or (mh "cardiomyopathy, hypertrophic") or (mh "cardiomyopathy, restrictive") or (mh "aortic valve disease") or ( mh "heart valve prolapse") or (mh "mitral valve insufficiency") or (mh "mitral valve stenosis") or (mh "pulmonary valve insufficiency") or (mh "pulmonary valve stenosis") or (mh "tricuspid valve insufficiency") or (mh "tricuspid valve stenosis") or (mh "aortic valve stenosis") or (mh "Truncus Arteriosus") or (mh "Fontan Procedure")

#6 ("congenital heart disease" or "congenital heart defect*" or "cyanotic heart disease" or "cyanotic heart defect*" or "p?ediatric heart disease" or "aortic valve stenosis" or "aortic atresia" or "quadricuspid aortic valve" or "bicuspid aortic valve" or "atrial septal defect*" or "aortic coarctation" or "coarctation of the aorta" or "hypoplastic aortic arch" or "interrupted aortic arch" or "atrioventricular canal" or "atrioventricular tunnel" or "atrioventricular septal defect" or "dextro transposition of the great arteries" or "levo transposition of the great arteries" or "transposition of the great arteries" or "transposition of the great vessels" or "Ebstein* anomaly" or "patent ductus arteriosus" or "patent foramen ovale" or "pulmonary valve stenosis" or "Tetralogy of Fallot" or "Trilogy of Fallot" or "Truncus Arteriosus" or hemitruncus or "single ventricle" or "univentricular heart" or "anomalous pulmonary venous" or "Scimitar syndrome" or "pulmonary venous stenosis" or "ventricular septal defect*" or "hypoplastic left heart syndrome" or "pulmonary atresia" or "pulmonary stenosis" or "tricuspid atresia" or "tricuspid stenosis" or "double outlet right ventricle" or "double inlet left ventricle" or "mitral atresia" or "mitral stenosis" or "congenital cardiomyopathy" or "p?ediatric cardiomyopathy" or "dilated cardiomyopathy" or "restricted cardiomyopathy" or " hypertrophic cardiomyopathy" or "arrhythmogenic right ventricular dysplasia" or "mitochondrial noncompaction" or "mitral valve disease" or "absent pulmonary valve" or "hypoplastic right heart syndrome" or Fontan or "vascular ring" or "Cor Triatriatum" or "pulmonary artery stenosis" or "pulmonary artery sling" or "double aortic arch" or "absent heart valve" or "Shone* complex*" or "aortic insufficiency" or "tricuspid insufficiency" or "mitral insufficiency" or "pulmonary insufficiency" or "pulmonary regurgitation" or "tricuspid regurgitation" or "mitral regurgitation" or "aortic regurgitation" or dextrocardia or "crisscross heart" or "Eisenmenger complex" or "Eisenmenger Syndrome" or "Ectopia Cordis" or "atrial isomerism*"):ti,ab,kw

#7 (mh "psychosocial Intervention") or (mh "psychosocial functioning") or (mh "psychosocial support systems")

#8 (psychosocial or psycho-social):ti,ab,kw

#9 (mh "adaptation, psychological") or (mh "attitude) or (mh "attitude to death") or (mh "attitude to health") or ( mh "catastrophization") or (mh optimism) or (mh pessimism) or (mh respect) or (mh stereotyping) or (mh "wit and humor as topic") or (mh behavior) or (mh "child rearing") or (mh "defense mechanisms") or (mh emotions) or (mh "human development") or (mh "mental competency") or (mh motivation) or (mh "neurobehavioral manifestations) or (mh personality) or (mh "psychology, social") or (mh temperance) or (mh "psychological phenomena") or (mh "mental disorders") or (mh "physical endurance) or (mh "physical exertion) or (mh "physical fitness") or (mh Fatigue) or (mh alcoholism) or (mh "substance abuse, intravenous") or (mh "substance abuse, oral")

#10 ("lived experience*" or "live through" or attitude* or psych* or mental* or "mental process*" or "mental health" or "mental disorder*" or mind* or feel* or thought* or emotion* or "social cognition*" or "cognitive process*" or depression or behavio?r* or anxiety or anxious or "attention deficit" or alcoholi* or lifestyle or suicid* or "cognitive deficit*" or "self-esteem" or "self-concept" or "body image" or "body perception" or fear* or "stress disorder" or anger or distrust or "medical trauma" or "physical trauma" or addict* or panic or "sexual activit*" or "sex life" or "sex drive" or "drug use" or "drug abuse*" or grief or griev* or bereave* or wellness or wellbeing or well-being or "physical health" or "physical shape" or "physical condition*" or "physical fitness*" or endurance or fatigue or "exercise tolerance" or "coping skill*" or "coping strateg*"):ti,ab,kw

#11 (mh "social justice") or (mh "public opinion") or (mh "social problems") or (mh "adverse childhood experiences") or (mh bullying) or (mh "dangerous behavior") or (mh divorce) or (mh "emotional abuse") or (mh poverty) or (mh "social segregation") or (mh suicide) or (mh "underage drinking") or (mh "social structure") or (mh "social welfare") or (mh "community resources") or (mh culture) or (mh "ethnic and racial minorities") or (mh "family relations") or (mh "family separation") or (mh "hierarchy, social") or (mh medicalization) or (mh "minority groups") or (mh secularism) or (mh "social capital") or (mh "social conditions") or (mh "social environment") or (mh "social isolation") or (mh "social marginalization") or (mh "social norms") or (mh "social vulnerability") or (mh "sociodemographic factors") or (mh "socioeconomic factors") or (mh "needs assessment") or (mh "return to school") or (mh "culturally competent care") or (mh "delivery of health care, integrated") or (mh "health services accessibility") or (mh "healthcare disparities") or (mh "health inequities") or (mh "educational status") 1461

#12 (friendship* or "family relation*" or "personal relationship*" or "interpersonal relationship*" or "social environment*" or "social factor*" or "social barrier*" or "social inclusion" or "social network*" or employer* or "social group*" or "social relationship*" or "professional relationship*" or "intimate relationship*" or "sexual partner" or "marital status" or "social structure*" or "society as a whole" or "socioeconomic status" or "political system*" or "judicial system*" or "legal system*" or "health* system*" or "health care system*" or "healthcare system*" or "health* program*" or "health care program*" or "healthcare group*" or "health care group*" or "education system*" or "school system" or "cultural environment*" or patriarchy or marginali* or discriminat* or judgement or "social isolation" or prejudice* or bias* or bigotry or intolerance* or "narrow mindedness" or inequalit* or inequit* or disadvantage* or disempower* or empower* or stigma* or vulnerable or bullying or bullied or oppress* or persecute* or "traumatic event*" or workplace* or "work place*OR social justice" or "social injustice*"):ti,ab,kw

#13 (mh employment) or (mh "employment, supported") or (mh unemployment) or (mh workplace) or (mh income)

#14 (finance* or income or "finance* service*" or "employ* service*" or "finance* resource* " or "employ* resource*" or "material resource*" or employ* or unemploy* or job? or "distribution of material resource*" or "social* disadvantage*" or "neo material" or money or "pecuniary fund*" or bankrupt* or welfare or "socioeconomic factor*"):ti,ab,kw

#15 (mh religion) or ( mh "religion and psychology") or (mh spirituality)

#16 (spiritual* or religion* or religious* or Ignatian or otherworldliness or unearthliness or incorporeality or piousness or devoutness or holiness or religiosity or pietism or piety or reverence or transcendence or "relationship to the transcendent" or "religious affiliation*"):ti,ab,kw

#17 (mh "Developed Countries")

#18 ("high income countr*" or "high-income countr*" or "high income nation*" or "high-income nation*" or "developed countr*" or "developed nation*" or "first world countr*" or "first world nation*"):ti,ab,kw

#19 (mh aruba) or (mh curacao) or (mh "sint maarten") or (mh "north america") or (mh canada) or (mh greenland) or (mh "united states") or (mh chile) or (mh uruguay) or (mh brunei) or (mh singapore) or (mh bahrain) or (mh israel) or (mh kuwait) or (mh oman) or (mh qatar) or (mh "saudi arabia") or (mh "united arab emirates") or (mh "hong kong") or (mh macau) or (mh japan) or (mh korea) or (mh taiwan) or (mh europe) or (mh andorra) or (mh austria) or (mh belgium) or (mh estonia) or (mh latvia) or (mh lithuania) or (mh croatia) or (mh czech republic) or (mh hungary) or (mh poland) or (mh slovakia) or (mh slovenia) or (mh france) or (mh germany) or (mh gibraltar) or (mh "united kingdom") or (mh "channel islands") or (mh england) or (mh "northern ireland") or (mh greece) or (mh ireland) or (mh italy) or (mh liechtenstein) or (mh luxembourg) or (mh monaco) or (mh netherlands) or (mh portugal) or (mh "san marino") or (mh denmark) or (mh finland) or (mh iceland) or (mh norway) or (mh sweden) or (mh spain) or (mh switzerland) or (mh australia) or (mh seychelles) or (mh cyprus) or (mh malta) or (mh "new zealand") or (mh polynesia) or (mh "antigua and barbuda") or (mh bahamas) or (mh "puerto rico") or (mh "saint kitts and nevis") or (mh "trinidad and tobago") or (mh "united states virgin islands") or (mh "British Virgin Islands") or (mh "new caledonia") or (mh guam) or (mh palau)

#20 (Andorra* or "Antiqua and Barbuda" or Antigua* or Barbuda* or Aruba* or Australia* or Austria* or Baham* or Bahrain* or Belgium or Belgian* or Bermud* or "British Virgin Island*" or "Brunei Darussalam" or Bruneian* or Canad* or "Cayman Island*" or Caymanian* or "Channel Island*" or Chile* or Croatia* or Curacao* or Cyprus or Cypriot* or Czech* or "Czech Republic*" or Denmark or Danish or Dane or Estoni* or "Faroe Island*" or Faroese or Finland* or Finnish or Fin* or France or French or "French Polynesia*" or German* or Gibraltar* or Greece or Greek* or Greenland* or Guam* or "Hong Kong" or Hongkonger* or "Hong Konger" or "Hong Kongese" or Hungar* or Iceland* or Ireland or Irish or "Isle of Man" or Manx* or Israel* or Ital* or Japan* or Korea* or Kuwait* or Lativ* or Liechtenstein* or Lithuan* or Luxembourg* or Macao or Macanese or Malta or Maltese or Maltin* or Monaco or Monegasque* or Monacan or Nauru* or Netherland* or Dutch or "New Caledonia*" or "New Zealand*" or "Northern Mariana Island*" or Norway or Norwegian* or Oman* or Palau* or Poland or Polish or Portugal or Portuguese or "Puerto Ric*" or Qatar* or "San Mariano" or Sammarinese or "Saudi Arabia*" or Saudi or Seychell* or Singapore* or "Sint Maarten*" or "Slovak Republic" or Slovak* or Slovenia* or Spain or Spaniard* or "St. Kitts and Nevis" or Kittitian* or Nevisian* or "St Martin*" or "Saint Martin*" or "Saint-Martin*" or Swed* or Switzerland or Swiss or Taiwan* or "Trinidad and Tobago" or Trinid* or Tobago* or "Turks and Caicos Island*" or "United Arab Emirates" or Emirati* or "United Kingdom" or England* or "British" or "United States" or American* or Uruguay* or "Virgin Island*" or "North Americ*" or Europe*):ti,ab,kw

#21 #1 OR #2 OR #3 OR #4

#22 #5 OR #6

#23 #7 OR #8 OR #9 OR #10 OR #11 OR #12 OR #13 OR #14 OR #15 OR #16

#24 #17 OR #18 OR #19 OR #20

#25 #21 AND #22 AND #23 AND #24

#26 #21 AND #22 AND #23 AND #24 with Publication Year from 2000 to present, in Trials

**Scopus Search Strategy**

S1

TITLE-ABS-KEY ( child* OR adolesc* OR teen* OR preteen* OR pre-teen* OR tween* OR juvenile* OR youth* OR minor* OR kid* OR "school age" OR schoolchild* OR "school child*" OR toddler* OR infan* OR neonat* OR baby OR babies OR newborn* OR "new born*" OR p#ediatric* OR prepubesc* OR pre-pubesc* OR pubescen* )

S2

TITLE-ABS-KEY ( caregiver* OR "care giver" OR carer* OR caretaker* OR "care taker*" OR guardian* OR parent* OR stepparent OR mom* OR mother* OR father* OR dad* OR stepfather* OR stepmother* OR grandparent* OR grandm* OR grandpa* OR grandfather* OR sibling* OR sister* OR brother* )

S3

TITLE-ABS-KEY ( "congenital heart disease" OR "congenital heart defect*" OR "cyanotic heart disease" OR "cyanotic heart defect*" OR "p#ediatric heart disease" OR "aortic valve stenosis" OR "aortic atresia" OR "quadricuspid aortic valve" OR "bicuspid aortic valve" OR "atrial septal defect*" OR "aortic coarctation" OR "coarctation of the aorta" OR "hypoplastic aortic arch" OR "interrupted aortic arch" OR "atrioventricular canal" OR "atrioventricular tunnel" OR "atrioventricular septal defect" OR "dextro transposition of the great arteries" OR "levo transposition of the great arteries" OR "transposition of the great arteries" OR "transposition of the great vessels" OR "ebstein* anomaly" OR "patent ductus arteriosus" OR "patent foramen ovale" OR "pulmonary valve stenosis" OR "tetralogy of fallot" OR "trilogy of fallot" OR "truncus arteriosus" OR hemitruncus OR "single ventricle" OR "univentricular heart" OR "anomalous pulmonary venous" OR "scimitar syndrome" OR "pulmonary venous stenosis" OR "ventricular septal defect*" OR "hypoplastic left heart syndrome" OR "pulmonary atresia" OR "pulmonary stenosis" OR "tricuspid atresia" OR "tricuspid stenosis" OR "double outlet right ventricle" OR "double inlet left ventricle" OR "mitral atresia" OR "mitral stenosis" OR "congenital cardiomyopathy" OR "p#ediatric cardiomyopathy" OR "dilated cardiomyopathy" OR "restricted cardiomyopathy" OR "hypertrophic cardiomyopathy" OR "arrhythmogenic right ventricular dysplasia" OR "mitochondrial noncompaction" OR "mitral valve disease" OR "absent pulmonary valve" OR "hypoplastic right heart syndrome" OR fontan OR "vascular ring" OR "cor triatriatum" OR "pulmonary artery stenosis" OR "pulmonary artery sling" OR "double aortic arch" OR "absent heart valve" OR "shone* complex*" OR "aortic insufficiency" OR "tricuspid insufficiency" OR "mitral insufficiency" OR "pulmonary insufficiency" OR "pulmonary regurgitation" OR "tricuspid regurgitation" OR "mitral regurgitation" OR "aortic regurgitation" OR dextrocardia OR "crisscross heart" OR "eisenmenger complex" OR "eisenmenger syndrome" OR "ectopia cordis" OR "atrial isomerism*" )

S4

TITLE-ABS-KEY ( psychosocial OR psycho-social )

S5

TITLE-ABS-KEY ( "lived experience*" OR "live through" OR attitude* OR psych* OR mental* OR "mental process*" OR "mental health" OR "mental disorder*" OR mind* OR feel* OR thought* OR emotion* OR "social cognition*" OR "cognitive process*" OR depression OR behavio#r* OR anxiety OR anxious OR "attention deficit" OR alcoholi* OR lifestyle OR suicid* OR "cognitive deficit*" OR "self-esteem" OR "self-concept" OR "body image" OR "body perception" OR fear* OR "stress disorder" OR anger OR distrust OR "medical trauma" OR "physical trauma" OR addict* OR panic OR "sexual activit*" OR "sex life" OR "sex drive" OR "drug use" OR "drug abuse*" OR grief OR griev* OR bereave* OR wellness OR wellbeing OR well-being OR "physical health" OR "physical shape" OR "physical condition*" OR "physical fitness*" OR endurance OR fatigue OR "exercise tolerance" OR "coping skill*" OR "coping strateg*" )

S6

TITLE-ABS-KEY ( friendship* OR "family relation*" OR "personal relationship*" OR "interpersonal relationship*" OR "social environment*" OR "social factor*" OR "social barrier*" OR "social inclusion" OR "social network*" OR employer* OR "social group*" OR "social relationship*" OR "professional relationship*" OR "intimate relationship*" OR "sexual partner" OR "marital status" OR "social structure*" OR "society as a whole" OR "socioeconomic status" OR "political system*" OR "judicial system*" OR "legal system*" OR "health* system*" OR "health care system*" OR "healthcare system*" OR "health* program*" OR "health care program*" OR "healthcare group*" OR "health care group*" OR "education system*" OR "school system" OR "cultural environment*" OR patriarchy OR marginali* OR discriminat* OR judgement OR "social isolation" OR prejudice* OR bias* OR bigotry OR intolerance* OR "narrow mindedness" OR inequalit* OR inequit* OR disadvantage* OR disempower* OR empower* OR stigma* OR vulnerable OR bullying OR bullied OR oppress* OR persecute* OR "traumatic event*" OR workplace* OR "work place*" OR "social justice" OR "social injustice*" )

S7

TITLE-ABS-KEY ( finance* OR income OR "finance* service*" OR "employ* service*" OR "finance* resource*" OR "employ* resource*" OR "material resource*" OR employ* OR unemploy* OR job* OR "distribution of material resource*" OR "social* disadvantage*" OR "neo material" OR money OR "pecuniary fund*" OR bankrupt* OR welfare OR "socioeconomic factor*" )

S8

TITLE-ABS-KEY ( spiritual* OR religion* OR religious* OR ignatian OR otherworldliness OR unearthliness OR incorporeality OR piousness OR devoutness OR holiness OR religiosity OR pietism OR piety OR reverence OR transcendence OR "relationship to the transcendent" OR "religious affiliation*" )

S9

TITLE-ABS-KEY ( "high income countr*" OR "high-income countr*" OR "high income nation*" OR "high-income nation*" OR "developed countr*" OR "developed nation*" OR "first world countr*" OR "first world nation*" )

S10

TITLE-ABS-KEY ( andorra* OR "antiqua and barbuda" OR antigua* OR barbuda* OR aruba* OR australia* OR austria* OR baham* OR bahrain* OR belgium OR belgian* OR bermud* OR "british virgin island*" OR "brunei darussalam" OR bruneian* OR canad* OR "cayman island*" OR caymanian* OR "channel island*" OR chile* OR croatia* OR curacao* OR cyprus OR cypriot* OR czech* OR "czech republic*" OR denmark OR danish OR dane OR estoni* OR "faroe island*" OR faroese OR finland* OR finnish OR fin* OR france OR french OR "french polynesia*" OR german* OR gibraltar* OR greece OR greek* OR greenland* OR guam* OR "hong kong" OR hongkonger* OR "hong konger" OR "hong kongese" OR hungar* OR iceland* OR ireland OR irish OR "isle of man" OR manx* OR israel* OR ital* OR japan* OR korea* OR kuwait* OR lativ* OR liechtenstein* OR lithuan* OR luxembourg* OR macao OR macanese OR malta OR maltese OR maltin* OR monaco OR monegasque* OR monacan OR nauru* OR netherland* OR dutch OR "new caledonia*" OR "new zealand*" OR "northern mariana island*" OR norway OR norwegian* OR oman* OR palau* OR poland OR polish OR portugal OR portuguese OR "puerto ric*" OR qatar* OR "san mariano" OR sammarinese OR "saudi arabia*" OR saudi OR seychell* OR singapore* OR "sint maarten*" OR "slovak republic" OR slovak* OR slovenia* OR spain OR spaniard* OR "st. kitts and nevis" OR kittitian* OR nevisian* OR "st martin*" OR "saint martin*" OR "saint-martin*" OR swed* OR switzerland OR swiss OR taiwan* OR "trinidad and tobago" OR trinid* OR tobago* OR "turks and caicos island*" OR "united arab emirates" OR emirati* OR "united kingdom" OR england* OR "british" OR "united states" OR american* OR uruguay* OR "virgin island*" OR "north americ*" OR europe* )

S11

( TITLE-ABS-KEY ( child* OR adolesc* OR teen* OR preteen* OR pre-teen* OR tween* OR juvenile* OR youth* OR minor* OR kid* OR "school age" OR schoolchild* OR "school child*" OR toddler* OR infan* OR neonat* OR baby OR babies OR newborn* OR "new born*" OR p#ediatric* OR prepubesc* OR pre-pubesc* OR pubescen* ) ) OR ( TITLE-ABS-KEY ( caregiver* OR "care giver" OR carer* OR caretaker* OR "care taker*" OR guardian* OR parent* OR stepparent OR mom* OR mother* OR father* OR dad* OR stepfather* OR stepmother* OR grandparent* OR grandm* OR grandpa* OR grandfather* OR sibling* OR sister* OR brother* ) )

S12

( TITLE-ABS-KEY ( psychosocial OR psycho-social ) ) OR ( TITLE-ABS-KEY ( "lived experience*" OR "live through" OR attitude* OR psych* OR mental* OR "mental process*" OR "mental health" OR "mental disorder*" OR mind* OR feel* OR thought* OR emotion* OR "social cognition*" OR "cognitive process*" OR depression OR behavio#r* OR anxiety OR anxious OR "attention deficit" OR alcoholi* OR lifestyle OR suicid* OR "cognitive deficit*" OR "self-esteem" OR "self-concept" OR "body image" OR "body perception" OR fear* OR "stress disorder" OR anger OR distrust OR "medical trauma" OR "physical trauma" OR addict* OR panic OR "sexual activit*" OR "sex life" OR "sex drive" OR "drug use" OR "drug abuse*" OR grief OR griev* OR bereave* OR wellness OR wellbeing OR well-being OR "physical health" OR "physical shape" OR "physical condition*" OR "physical fitness*" OR endurance OR fatigue OR "exercise tolerance" OR "coping skill*" OR "coping strateg*" ) ) OR ( TITLE-ABS-KEY ( friendship* OR "family relation*" OR "personal relationship*" OR "interpersonal relationship*" OR "social environment*" OR "social factor*" OR "social barrier*" OR "social inclusion" OR "social network*" OR employer* OR "social group*" OR "social relationship*" OR "professional relationship*" OR "intimate relationship*" OR "sexual partner" OR "marital status" OR "social structure*" OR "society as a whole" OR "socioeconomic status" OR "political system*" OR "judicial system*" OR "legal system*" OR "health* system*" OR "health care system*" OR "healthcare system*" OR "health* program*" OR "health care program*" OR "healthcare group*" OR "health care group*" OR "education system*" OR "school system" OR "cultural environment*" OR patriarchy OR marginali* OR discriminat* OR judgement OR "social isolation" OR prejudice* OR bias* OR bigotry OR intolerance* OR "narrow mindedness" OR inequalit* OR inequit* OR disadvantage* OR disempower* OR empower* OR stigma* OR vulnerable OR bullying OR bullied OR oppress* OR persecute* OR "traumatic event*" OR workplace* OR "work place*" OR "social justice" OR "social injustice*" ) ) OR ( TITLE-ABS-KEY ( finance* OR income OR "finance* service*" OR "employ* service*" OR "finance* resource*" OR "employ* resource*" OR "material resource*" OR employ* OR unemploy* OR job* OR "distribution of material resource*" OR "social* disadvantage*" OR "neo material" OR money OR "pecuniary fund*" OR bankrupt* OR welfare OR "socioeconomic factor*" ) ) OR ( TITLE-ABS-KEY ( spiritual* OR religion* OR religious* OR ignatian OR otherworldliness OR unearthliness OR incorporeality OR piousness OR devoutness OR holiness OR religiosity OR pietism OR piety OR reverence OR transcendence OR "relationship to the transcendent" OR "religious affiliation*" ) )

S13

( TITLE-ABS-KEY ( "high income countr*" OR "high-income countr*" OR "high income nation*" OR "high-income nation*" OR "developed countr*" OR "developed nation*" OR "first world countr*" OR "first world nation*" ) ) OR ( TITLE-ABS-KEY ( andorra* OR "antiqua and barbuda" OR antigua* OR barbuda* OR aruba* OR australia* OR austria* OR baham* OR bahrain* OR belgium OR belgian* OR bermud* OR "british virgin island*" OR "brunei darussalam" OR bruneian* OR canad* OR "cayman island*" OR caymanian* OR "channel island*" OR chile* OR croatia* OR curacao* OR cyprus OR cypriot* OR czech* OR "czech republic*" OR denmark OR danish OR dane OR estoni* OR "faroe island*" OR faroese OR finland* OR finnish OR fin* OR france OR french OR "french polynesia*" OR german* OR gibraltar* OR greece OR greek* OR greenland* OR guam* OR "hong kong" OR hongkonger* OR "hong konger" OR "hong kongese" OR hungar* OR iceland* OR ireland OR irish OR "isle of man" OR manx* OR israel* OR ital* OR japan* OR korea* OR kuwait* OR lativ* OR liechtenstein* OR lithuan* OR luxembourg* OR macao OR macanese OR malta OR maltese OR maltin* OR monaco OR monegasque* OR monacan OR nauru* OR netherland* OR dutch OR "new caledonia*" OR "new zealand*" OR "northern mariana island*" OR norway OR norwegian* OR oman* OR palau* OR poland OR polish OR portugal OR portuguese OR "puerto ric*" OR qatar* OR "san mariano" OR sammarinese OR "saudi arabia*" OR saudi OR seychell* OR singapore* OR "sint maarten*" OR "slovak republic" OR slovak* OR slovenia* OR spain OR spaniard* OR "st. kitts and nevis" OR kittitian* OR nevisian* OR "st martin*" OR "saint martin*" OR "saint-martin*" OR swed* OR switzerland OR swiss OR taiwan* OR "trinidad and tobago" OR trinid* OR tobago* OR "turks and caicos island*" OR "united arab emirates" OR emirati* OR "united kingdom" OR england* OR "british" OR "united states" OR american* OR uruguay* OR "virgin island*" OR "north americ*" OR europe* ) )

S14

( ( TITLE-ABS-KEY ( child* OR adolesc* OR teen* OR preteen* OR pre-teen* OR tween* OR juvenile* OR youth* OR minor* OR kid* OR "school age" OR schoolchild* OR "school child*" OR toddler* OR infan* OR neonat* OR baby OR babies OR newborn* OR "new born*" OR p#ediatric* OR prepubesc* OR pre-pubesc* OR pubescen* ) ) OR ( TITLE-ABS-KEY ( caregiver* OR "care giver" OR carer* OR caretaker* OR "care taker*" OR guardian* OR parent* OR stepparent OR mom* OR mother* OR father* OR dad* OR stepfather* OR stepmother* OR grandparent* OR grandm* OR grandpa* OR grandfather* OR sibling* OR sister* OR brother* ) ) ) AND ( TITLE-ABS-KEY ( "congenital heart disease" OR "congenital heart defect*" OR "cyanotic heart disease" OR "cyanotic heart defect*" OR "p#ediatric heart disease" OR "aortic valve stenosis" OR "aortic atresia" OR "quadricuspid aortic valve" OR "bicuspid aortic valve" OR "atrial septal defect*" OR "aortic coarctation" OR "coarctation of the aorta" OR "hypoplastic aortic arch" OR "interrupted aortic arch" OR "atrioventricular canal" OR "atrioventricular tunnel" OR "atrioventricular septal defect" OR "dextro transposition of the great arteries" OR "levo transposition of the great arteries" OR "transposition of the great arteries" OR "transposition of the great vessels" OR "ebstein* anomaly" OR "patent ductus arteriosus" OR "patent foramen ovale" OR "pulmonary valve stenosis" OR "tetralogy of fallot" OR "trilogy of fallot" OR "truncus arteriosus" OR hemitruncus OR "single ventricle" OR "univentricular heart" OR "anomalous pulmonary venous" OR "scimitar syndrome" OR "pulmonary venous stenosis" OR "ventricular septal defect*" OR "hypoplastic left heart syndrome" OR "pulmonary atresia" OR "pulmonary stenosis" OR "tricuspid atresia" OR "tricuspid stenosis" OR "double outlet right ventricle" OR "double inlet left ventricle" OR "mitral atresia" OR "mitral stenosis" OR "congenital cardiomyopathy" OR "p#ediatric cardiomyopathy" OR "dilated cardiomyopathy" OR "restricted cardiomyopathy" OR "hypertrophic cardiomyopathy" OR "arrhythmogenic right ventricular dysplasia" OR "mitochondrial noncompaction" OR "mitral valve disease" OR "absent pulmonary valve" OR "hypoplastic right heart syndrome" OR fontan OR "vascular ring" OR "cor triatriatum" OR "pulmonary artery stenosis" OR "pulmonary artery sling" OR "double aortic arch" OR "absent heart valve" OR "shone* complex*" OR "aortic insufficiency" OR "tricuspid insufficiency" OR "mitral insufficiency" OR "pulmonary insufficiency" OR "pulmonary regurgitation" OR "tricuspid regurgitation" OR "mitral regurgitation" OR "aortic regurgitation" OR dextrocardia OR "crisscross heart" OR "eisenmenger complex" OR "eisenmenger syndrome" OR "ectopia cordis" OR "atrial isomerism*" ) ) AND ( ( TITLE-ABS-KEY ( psychosocial OR psycho-social ) ) OR ( TITLE-ABS-KEY ( "lived experience*" OR "live through" OR attitude* OR psych* OR mental* OR "mental process*" OR "mental health" OR "mental disorder*" OR mind* OR feel* OR thought* OR emotion* OR "social cognition*" OR "cognitive process*" OR depression OR behavio#r* OR anxiety OR anxious OR "attention deficit" OR alcoholi* OR lifestyle OR suicid* OR "cognitive deficit*" OR "self-esteem" OR "self-concept" OR "body image" OR "body perception" OR fear* OR "stress disorder" OR anger OR distrust OR "medical trauma" OR "physical trauma" OR addict* OR panic OR "sexual activit*" OR "sex life" OR "sex drive" OR "drug use" OR "drug abuse*" OR grief OR griev* OR bereave* OR wellness OR wellbeing OR well-being OR "physical health" OR "physical shape" OR "physical condition*" OR "physical fitness*" OR endurance OR fatigue OR "exercise tolerance" OR "coping skill*" OR "coping strateg*" ) ) OR ( TITLE-ABS-KEY ( friendship* OR "family relation*" OR "personal relationship*" OR "interpersonal relationship*" OR "social environment*" OR "social factor*" OR "social barrier*" OR "social inclusion" OR "social network*" OR employer* OR "social group*" OR "social relationship*" OR "professional relationship*" OR "intimate relationship*" OR "sexual partner" OR "marital status" OR "social structure*" OR "society as a whole" OR "socioeconomic status" OR "political system*" OR "judicial system*" OR "legal system*" OR "health* system*" OR "health care system*" OR "healthcare system*" OR "health* program*" OR "health care program*" OR "healthcare group*" OR "health care group*" OR "education system*" OR "school system" OR "cultural environment*" OR patriarchy OR marginali* OR discriminat* OR judgement OR "social isolation" OR prejudice* OR bias* OR bigotry OR intolerance* OR "narrow mindedness" OR inequalit* OR inequit* OR disadvantage* OR disempower* OR empower* OR stigma* OR vulnerable OR bullying OR bullied OR oppress* OR persecute* OR "traumatic event*" OR workplace* OR "work place*" OR "social justice" OR "social injustice*" ) ) OR ( TITLE-ABS-KEY ( finance* OR income OR "finance* service*" OR "employ* service*" OR "finance* resource*" OR "employ* resource*" OR "material resource*" OR employ* OR unemploy* OR job* OR "distribution of material resource*" OR "social* disadvantage*" OR "neo material" OR money OR "pecuniary fund*" OR bankrupt* OR welfare OR "socioeconomic factor*" ) ) OR ( TITLE-ABS-KEY ( spiritual* OR religion* OR religious* OR ignatian OR otherworldliness OR unearthliness OR incorporeality OR piousness OR devoutness OR holiness OR religiosity OR pietism OR piety OR reverence OR transcendence OR "relationship to the transcendent" OR "religious affiliation*" ) ) ) AND ( ( TITLE-ABS-KEY ( "high income countr*" OR "high-income countr*" OR "high income nation*" OR "high-income nation*" OR "developed countr*" OR "developed nation*" OR "first world countr*" OR "first world nation*" ) ) OR ( TITLE-ABS-KEY ( andorra* OR "antiqua and barbuda" OR antigua* OR barbuda* OR aruba* OR australia* OR austria* OR baham* OR bahrain* OR belgium OR belgian* OR bermud* OR "british virgin island*" OR "brunei darussalam" OR bruneian* OR canad* OR "cayman island*" OR caymanian* OR "channel island*" OR chile* OR croatia* OR curacao* OR cyprus OR cypriot* OR czech* OR "czech republic*" OR denmark OR danish OR dane OR estoni* OR "faroe island*" OR faroese OR finland* OR finnish OR fin* OR france OR french OR "french polynesia*" OR german* OR gibraltar* OR greece OR greek* OR greenland* OR guam* OR "hong kong" OR hongkonger* OR "hong konger" OR "hong kongese" OR hungar* OR iceland* OR ireland OR irish OR "isle of man" OR manx* OR israel* OR ital* OR japan* OR korea* OR kuwait* OR lativ* OR liechtenstein* OR lithuan* OR luxembourg* OR macao OR macanese OR malta OR maltese OR maltin* OR monaco OR monegasque* OR monacan OR nauru* OR netherland* OR dutch OR "new caledonia*" OR "new zealand*" OR "northern mariana island*" OR norway OR norwegian* OR oman* OR palau* OR poland OR polish OR portugal OR portuguese OR "puerto ric*" OR qatar* OR "san mariano" OR sammarinese OR "saudi arabia*" OR saudi OR seychell* OR singapore* OR "sint maarten*" OR "slovak republic" OR slovak* OR slovenia* OR spain OR spaniard* OR "st. kitts and nevis" OR kittitian* OR nevisian* OR "st martin*" OR "saint martin*" OR "saint-martin*" OR swed* OR switzerland OR swiss OR taiwan* OR "trinidad and tobago" OR trinid* OR tobago* OR "turks and caicos island*" OR "united arab emirates" OR emirati* OR "united kingdom" OR england* OR "british" OR "united states" OR american* OR uruguay* OR "virgin island*" OR "north americ*" OR europe* ) ) )

S15

( ( TITLE-ABS-KEY ( child* OR adolesc* OR teen* OR preteen* OR pre-teen* OR tween* OR juvenile* OR youth* OR minor* OR kid* OR "school age" OR schoolchild* OR "school child*" OR toddler* OR infan* OR neonat* OR baby OR babies OR newborn* OR "new born*" OR p#ediatric* OR prepubesc* OR pre-pubesc* OR pubescen* ) ) OR ( TITLE-ABS-KEY ( caregiver* OR "care giver" OR carer* OR caretaker* OR "care taker*" OR guardian* OR parent* OR stepparent OR mom* OR mother* OR father* OR dad* OR stepfather* OR stepmother* OR grandparent* OR grandm* OR grandpa* OR grandfather* OR sibling* OR sister* OR brother* ) ) ) AND ( TITLE-ABS-KEY ( "congenital heart disease" OR "congenital heart defect*" OR "cyanotic heart disease" OR "cyanotic heart defect*" OR "p#ediatric heart disease" OR "aortic valve stenosis" OR "aortic atresia" OR "quadricuspid aortic valve" OR "bicuspid aortic valve" OR "atrial septal defect*" OR "aortic coarctation" OR "coarctation of the aorta" OR "hypoplastic aortic arch" OR "interrupted aortic arch" OR "atrioventricular canal" OR "atrioventricular tunnel" OR "atrioventricular septal defect" OR "dextro transposition of the great arteries" OR "levo transposition of the great arteries" OR "transposition of the great arteries" OR "transposition of the great vessels" OR "ebstein* anomaly" OR "patent ductus arteriosus" OR "patent foramen ovale" OR "pulmonary valve stenosis" OR "tetralogy of fallot" OR "trilogy of fallot" OR "truncus arteriosus" OR hemitruncus OR "single ventricle" OR "univentricular heart" OR "anomalous pulmonary venous" OR "scimitar syndrome" OR "pulmonary venous stenosis" OR "ventricular septal defect*" OR "hypoplastic left heart syndrome" OR "pulmonary atresia" OR "pulmonary stenosis" OR "tricuspid atresia" OR "tricuspid stenosis" OR "double outlet right ventricle" OR "double inlet left ventricle" OR "mitral atresia" OR "mitral stenosis" OR "congenital cardiomyopathy" OR "p#ediatric cardiomyopathy" OR "dilated cardiomyopathy" OR "restricted cardiomyopathy" OR "hypertrophic cardiomyopathy" OR "arrhythmogenic right ventricular dysplasia" OR "mitochondrial noncompaction" OR "mitral valve disease" OR "absent pulmonary valve" OR "hypoplastic right heart syndrome" OR fontan OR "vascular ring" OR "cor triatriatum" OR "pulmonary artery stenosis" OR "pulmonary artery sling" OR "double aortic arch" OR "absent heart valve" OR "shone* complex*" OR "aortic insufficiency" OR "tricuspid insufficiency" OR "mitral insufficiency" OR "pulmonary insufficiency" OR "pulmonary regurgitation" OR "tricuspid regurgitation" OR "mitral regurgitation" OR "aortic regurgitation" OR dextrocardia OR "crisscross heart" OR "eisenmenger complex" OR "eisenmenger syndrome" OR "ectopia cordis" OR "atrial isomerism*" ) ) AND ( ( TITLE-ABS-KEY ( psychosocial OR psycho-social ) ) OR ( TITLE-ABS-KEY ( "lived experience*" OR "live through" OR attitude* OR psych* OR mental* OR "mental process*" OR "mental health" OR "mental disorder*" OR mind* OR feel* OR thought* OR emotion* OR "social cognition*" OR "cognitive process*" OR depression OR behavio#r* OR anxiety OR anxious OR "attention deficit" OR alcoholi* OR lifestyle OR suicid* OR "cognitive deficit*" OR "self-esteem" OR "self-concept" OR "body image" OR "body perception" OR fear* OR "stress disorder" OR anger OR distrust OR "medical trauma" OR "physical trauma" OR addict* OR panic OR "sexual activit*" OR "sex life" OR "sex drive" OR "drug use" OR "drug abuse*" OR grief OR griev* OR bereave* OR wellness OR wellbeing OR well-being OR "physical health" OR "physical shape" OR "physical condition*" OR "physical fitness*" OR endurance OR fatigue OR "exercise tolerance" OR "coping skill*" OR "coping strateg*" ) ) OR ( TITLE-ABS-KEY ( friendship* OR "family relation*" OR "personal relationship*" OR "interpersonal relationship*" OR "social environment*" OR "social factor*" OR "social barrier*" OR "social inclusion" OR "social network*" OR employer* OR "social group*" OR "social relationship*" OR "professional relationship*" OR "intimate relationship*" OR "sexual partner" OR "marital status" OR "social structure*" OR "society as a whole" OR "socioeconomic status" OR "political system*" OR "judicial system*" OR "legal system*" OR "health* system*" OR "health care system*" OR "healthcare system*" OR "health* program*" OR "health care program*" OR "healthcare group*" OR "health care group*" OR "education system*" OR "school system" OR "cultural environment*" OR patriarchy OR marginali* OR discriminat* OR judgement OR "social isolation" OR prejudice* OR bias* OR bigotry OR intolerance* OR "narrow mindedness" OR inequalit* OR inequit* OR disadvantage* OR disempower* OR empower* OR stigma* OR vulnerable OR bullying OR bullied OR oppress* OR persecute* OR "traumatic event*" OR workplace* OR "work place*" OR "social justice" OR "social injustice*" ) ) OR ( TITLE-ABS-KEY ( finance* OR income OR "finance* service*" OR "employ* service*" OR "finance* resource*" OR "employ* resource*" OR "material resource*" OR employ* OR unemploy* OR job* OR "distribution of material resource*" OR "social* disadvantage*" OR "neo material" OR money OR "pecuniary fund*" OR bankrupt* OR welfare OR "socioeconomic factor*" ) ) OR ( TITLE-ABS-KEY ( spiritual* OR religion* OR religious* OR ignatian OR otherworldliness OR unearthliness OR incorporeality OR piousness OR devoutness OR holiness OR religiosity OR pietism OR piety OR reverence OR transcendence OR "relationship to the transcendent" OR "religious affiliation*" ) ) ) AND ( ( TITLE-ABS-KEY ( "high income countr*" OR "high-income countr*" OR "high income nation*" OR "high-income nation*" OR "developed countr*" OR "developed nation*" OR "first world countr*" OR "first world nation*" ) ) OR ( TITLE-ABS-KEY ( andorra* OR "antiqua and barbuda" OR antigua* OR barbuda* OR aruba* OR australia* OR austria* OR baham* OR bahrain* OR belgium OR belgian* OR bermud* OR "british virgin island*" OR "brunei darussalam" OR bruneian* OR canad* OR "cayman island*" OR caymanian* OR "channel island*" OR chile* OR croatia* OR curacao* OR cyprus OR cypriot* OR czech* OR "czech republic*" OR denmark OR danish OR dane OR estoni* OR "faroe island*" OR faroese OR finland* OR finnish OR fin* OR france OR french OR "french polynesia*" OR german* OR gibraltar* OR greece OR greek* OR greenland* OR guam* OR "hong kong" OR hongkonger* OR "hong konger" OR "hong kongese" OR hungar* OR iceland* OR ireland OR irish OR "isle of man" OR manx* OR israel* OR ital* OR japan* OR korea* OR kuwait* OR lativ* OR liechtenstein* OR lithuan* OR luxembourg* OR macao OR macanese OR malta OR maltese OR maltin* OR monaco OR monegasque* OR monacan OR nauru* OR netherland* OR dutch OR "new caledonia*" OR "new zealand*" OR "northern mariana island*" OR norway OR norwegian* OR oman* OR palau* OR poland OR polish OR portugal OR portuguese OR "puerto ric*" OR qatar* OR "san mariano" OR sammarinese OR "saudi arabia*" OR saudi OR seychell* OR singapore* OR "sint maarten*" OR "slovak republic" OR slovak* OR slovenia* OR spain OR spaniard* OR "st. kitts and nevis" OR kittitian* OR nevisian* OR "st martin*" OR "saint martin*" OR "saint-martin*" OR swed* OR switzerland OR swiss OR taiwan* OR "trinidad and tobago" OR trinid* OR tobago* OR "turks and caicos island*" OR "united arab emirates" OR emirati* OR "united kingdom" OR england* OR "british" OR "united states" OR american* OR uruguay* OR "virgin island*" OR "north americ*" OR europe* ) ) ) AND PUBYEAR > 1999
