# Supplementary material for: An examination of the psychosocial consequences experienced by children and adolescents living with congenital heart disease and their primary caregivers: a scoping review protocol
Source: Syst Rev. 2023 Jun 2;12:90. doi: 10.1186/s13643-023-02249-7 (PMC10239103; doi:10.1186/s13643-023-02249-7)
Supplement: Supplementary file 6 — Additional file 6. Search strategies for grey literature. [file 13643_2023_2249_MOESM6_ESM.docx]

**Additonal file 6**

**Search strategies for grey literature**

**ProQuest Theses and Dissertations Global Search Strategy**

| **S1** | noft(child* OR adolesc* OR teen* OR preteen* OR pre-teen* OR toddler* OR infan* OR neonat* OR baby OR babies OR newborn* OR "new born*" OR p?ediatric* OR caregiver* OR "care giver" OR guardian* OR parent* OR stepparent OR mother* OR father* OR stepfather* OR stepmother* OR grandparent* OR grandm* OR grandpa* OR grandfather* OR sibling*)  Databases:  ProQuest Dissertations & Theses Global |
| --- | --- |
| **S2** | [noft("congenital heart disease" OR "congenital heart defect*" OR (aortic PRE/2 (stenosis or atresia or insufficiency or regurgitation)) OR (pulmonary PRE/2 (stenosis or atresia or insufficiency or regurgitation)) OR (tricuspid PRE/2 (stenosis or atresia or insufficiency or regurgitation)) OR (mitral PRE/2 (stenosis or atresia or insufficiency or regurgitation)) OR "atrial septal defect*" OR "coarctation of the aorta" OR "hypoplastic aortic arch" OR "interrupted aortic arch" OR "atrioventricular" OR "transposition of the great arteries" OR "transposition of the great vessels" OR "Ebstein* anomaly" OR "patent ductus arteriosus" OR "patent foramen ovale" OR "Tetralogy of Fallot" OR "Trilogy of Fallot" OR "Truncus Arteriosus" OR "single ventricle" OR "univentricular heart" OR "anomalous pulmonary venous" OR "Scimitar syndrome" OR "ventricular septal defect*" OR "hypoplastic left heart syndrome" OR "double outlet right ventricle" OR "double inlet left ventricle" OR "cardiomyopathy" OR "hypoplastic right heart syndrome" OR Fontan OR "vascular ring" OR "Cor Triatriatum" OR "double aortic arch" OR "absent heart valve" OR "Shone* complex*" OR dextrocardia OR Eisenmenger OR "Ectopia Cordis" OR "atrial isomerism*")](https://www.proquest.com/recentsearches.recentsearchtabview.recentsearchesgridview.scrolledrecentsearchlist.checkdbssearchlink:rerunsearch/7662F5CCFA740C4PQ/None?site=pqdtglobal&t:ac=RecentSearches)  Databases:  ProQuest Dissertations & Theses Global |
| **S3** | [noft(psychosocial OR "lived experience*" OR attitude* OR psych* OR mental* OR mind* OR feel* OR thought* OR emotion* OR "social cognition*" OR cognitive OR depression OR behavio?r* OR anxiety OR anxious OR "attention deficit" OR alcoholi* OR lifestyle OR suicid* OR "self-esteem" OR "self-concept" OR "body image" OR "body perception" OR fear* OR stress OR anger OR distrust OR trauma OR addict* OR panic OR "sexual activit*" OR "drug abuse*" OR grief OR griev* OR bereave* OR wellness OR wellbeing OR well-being OR "physical health" OR "physical shape" OR "physical condition*" OR "physical fitness*" OR endurance OR fatigue OR "exercise tolerance" OR "coping skill*" OR "coping strateg*" OR friendship* OR "family relation*" OR "personal relationship*" OR "interpersonal relationship*" OR "social inclusion" OR "social network*" OR "social group*" OR "marital status" OR "social structure*" OR "socioeconomic status" OR "political system*" OR "judicial system*" OR "legal system*" OR "health* system*" OR "health* program*" OR "health* group*" OR "education system*" OR "school system" OR cultural OR patriarchy OR marginali* OR discriminat* OR judgement OR "social isolation" OR prejudice* OR bias* OR bigotry OR intolerance*OR inequalit* OR inequit* OR disadvantage* OR disempower* OR empower* OR stigma* OR vulnerable OR bullying OR bullied OR oppress* OR persecute* OR "traumatic event*" OR workplace* OR "work place*"OR "social justice" OR "social injustice*" OR finance* OR income OR employ* OR unemploy* OR job* OR "social* disadvantage*" OR welfare OR "socioeconomic factor*" OR spiritual* OR religion* OR religious* OR religiosity OR "religious affiliation*" )](https://www.proquest.com/recentsearches.recentsearchtabview.recentsearchesgridview.scrolledrecentsearchlist.checkdbssearchlink:rerunsearch/93CD95C4726B46D8PQ/None?site=pqdtglobal&t:ac=RecentSearches)  Databases:  ProQuest Dissertations & Theses Global |
| **S4** | [noft(child* OR adolesc* OR teen* OR preteen* OR pre-teen* OR toddler* OR infan* OR neonat* OR baby OR babies OR newborn* OR "new born*" OR p?ediatric* OR caregiver* OR "care giver" OR guardian* OR parent* OR stepparent OR mother* OR father* OR stepfather* OR stepmother* OR grandparent* OR grandm* OR grandpa* OR grandfather* OR sibling*) AND noft("congenital heart disease" OR "congenital heart defect*" OR (aortic PRE/2 (stenosis OR atresia OR insufficiency OR regurgitation)) OR (pulmonary PRE/2 (stenosis OR atresia OR insufficiency OR regurgitation)) OR (tricuspid PRE/2 (stenosis OR atresia OR insufficiency OR regurgitation)) OR (mitral PRE/2 (stenosis OR atresia OR insufficiency OR regurgitation)) OR "atrial septal defect*" OR "coarctation of the aorta" OR "hypoplastic aortic arch" OR "interrupted aortic arch" OR "atrioventricular" OR "transposition of the great arteries" OR "transposition of the great vessels" OR "Ebstein* anomaly" OR "patent ductus arteriosus" OR "patent foramen ovale" OR "Tetralogy of Fallot" OR "Trilogy of Fallot" OR "Truncus Arteriosus" OR "single ventricle" OR "univentricular heart" OR "anomalous pulmonary venous" OR "Scimitar syndrome" OR "ventricular septal defect*" OR "hypoplastic left heart syndrome" OR "double outlet right ventricle" OR "double inlet left ventricle" OR "cardiomyopathy" OR "hypoplastic right heart syndrome" OR Fontan OR "vascular ring" OR "Cor Triatriatum" OR "double aortic arch" OR "absent heart valve" OR "Shone* complex*" OR dextrocardia OR Eisenmenger OR "Ectopia Cordis" OR "atrial isomerism*") AND noft(psychosocial OR "lived experience*" OR attitude* OR psych* OR mental* OR mind* OR feel* OR thought* OR emotion* OR "social cognition*" OR cognitive OR depression OR behavio?r* OR anxiety OR anxious OR "attention deficit" OR alcoholi* OR lifestyle OR suicid* OR "self-esteem" OR "self-concept" OR "body image" OR "body perception" OR fear* OR stress OR anger OR distrust OR trauma OR addict* OR panic OR "sexual activit*" OR "drug abuse*" OR grief OR griev* OR bereave* OR wellness OR wellbeing OR well-being OR "physical health" OR "physical shape" OR "physical condition*" OR "physical fitness*" OR endurance OR fatigue OR "exercise tolerance" OR "coping skill*" OR "coping strateg*" OR friendship* OR "family relation*" OR "personal relationship*" OR "interpersonal relationship*" OR "social inclusion" OR "social network*" OR "social group*" OR "marital status" OR "social structure*" OR "socioeconomic status" OR "political system*" OR "judicial system*" OR "legal system*" OR "health* system*" OR "health* program*" OR "health* group*" OR "education system*" OR "school system" OR cultural OR patriarchy OR marginali* OR discriminat* OR judgement OR "social isolation" OR prejudice* OR bias* OR bigotry OR intolerance*OR inequalit* OR inequit* OR disadvantage* OR disempower* OR empower* OR stigma* OR vulnerable OR bullying OR bullied OR oppress* OR persecute* OR "traumatic event*" OR workplace* OR "work place*" OR "social justice" OR "social injustice*" OR finance* OR income OR employ* OR unemploy* OR job* OR "social* disadvantage*" OR welfare OR "socioeconomic factor*" OR spiritual* OR religion* OR religious* OR religiosity OR "religious affiliation*")](https://www.proquest.com/recentsearches.recentsearchtabview.recentsearchesgridview.scrolledrecentsearchlist.checkdbssearchlink:rerunsearch/6BF536F9E9E24768PQ/None?site=pqdtglobal&t:ac=RecentSearches)  Databases:  ProQuest Dissertations & Theses Global |
| **S5** | [noft(child* OR adolesc* OR teen* OR preteen* OR pre-teen* OR toddler* OR infan* OR neonat* OR baby OR babies OR newborn* OR "new born*" OR p?ediatric* OR caregiver* OR "care giver" OR guardian* OR parent* OR stepparent OR mother* OR father* OR stepfather* OR stepmother* OR grandparent* OR grandm* OR grandpa* OR grandfather* OR sibling*) AND noft("congenital heart disease" OR "congenital heart defect*" OR (aortic PRE/2 (stenosis OR atresia OR insufficiency OR regurgitation)) OR (pulmonary PRE/2 (stenosis OR atresia OR insufficiency OR regurgitation)) OR (tricuspid PRE/2 (stenosis OR atresia OR insufficiency OR regurgitation)) OR (mitral PRE/2 (stenosis OR atresia OR insufficiency OR regurgitation)) OR "atrial septal defect*" OR "coarctation of the aorta" OR "hypoplastic aortic arch" OR "interrupted aortic arch" OR "atrioventricular" OR "transposition of the great arteries" OR "transposition of the great vessels" OR "Ebstein* anomaly" OR "patent ductus arteriosus" OR "patent foramen ovale" OR "Tetralogy of Fallot" OR "Trilogy of Fallot" OR "Truncus Arteriosus" OR "single ventricle" OR "univentricular heart" OR "anomalous pulmonary venous" OR "Scimitar syndrome" OR "ventricular septal defect*" OR "hypoplastic left heart syndrome" OR "double outlet right ventricle" OR "double inlet left ventricle" OR "cardiomyopathy" OR "hypoplastic right heart syndrome" OR Fontan OR "vascular ring" OR "Cor Triatriatum" OR "double aortic arch" OR "absent heart valve" OR "Shone* complex*" OR dextrocardia OR Eisenmenger OR "Ectopia Cordis" OR "atrial isomerism*") AND noft(psychosocial OR "lived experience*" OR attitude* OR psych* OR mental* OR mind* OR feel* OR thought* OR emotion* OR "social cognition*" OR cognitive OR depression OR behavio?r* OR anxiety OR anxious OR "attention deficit" OR alcoholi* OR lifestyle OR suicid* OR "self-esteem" OR "self-concept" OR "body image" OR "body perception" OR fear* OR stress OR anger OR distrust OR trauma OR addict* OR panic OR "sexual activit*" OR "drug abuse*" OR grief OR griev* OR bereave* OR wellness OR wellbeing OR well-being OR "physical health" OR "physical shape" OR "physical condition*" OR "physical fitness*" OR endurance OR fatigue OR "exercise tolerance" OR "coping skill*" OR "coping strateg*" OR friendship* OR "family relation*" OR "personal relationship*" OR "interpersonal relationship*" OR "social inclusion" OR "social network*" OR "social group*" OR "marital status" OR "social structure*" OR "socioeconomic status" OR "political system*" OR "judicial system*" OR "legal system*" OR "health* system*" OR "health* program*" OR "health* group*" OR "education system*" OR "school system" OR cultural OR patriarchy OR marginali* OR discriminat* OR judgement OR "social isolation" OR prejudice* OR bias* OR bigotry OR intolerance*OR inequalit* OR inequit* OR disadvantage* OR disempower* OR empower* OR stigma* OR vulnerable OR bullying OR bullied OR oppress* OR persecute* OR "traumatic event*" OR workplace* OR "work place*" OR "social justice" OR "social injustice*" OR finance* OR income OR employ* OR unemploy* OR job* OR "social* disadvantage*" OR welfare OR "socioeconomic factor*" OR spiritual* OR religion* OR religious* OR religiosity OR "religious affiliation*")](https://www.proquest.com/recentsearches.recentsearchtabview.recentsearchesgridview.scrolledrecentsearchlist.checkdbssearchlink:rerunsearch/77F25F5DD45A4445PQ/None?site=pqdtglobal&t:ac=RecentSearches)Limits applied  Databases:   - ProQuest Dissertations & Theses Global   Narrowed by:  Entered date:  2000-01-01 - 2022-05-29 |

**Search Strategy for Google Advanced Search**

1. child* AND "congenital heart disease "AND (psychosocial OR "psychosocial intervention*" OR "psychosocial support system*")
2. adoles* AND "congenital heart Disease "AND (psychosocial" OR psychosocial intervention*" OR "psychosocial support system*")
3. caregiver* AND "congenital heart Disease "AND (pyschosocial OR psychosocial intervention*" OR "psychosocial support system*")
4. parent* AND "congenital heart disease" AND (psychosocial OR psychosocial intervention*" OR "psychosocial support system*")
