# Supplementary material for: An examination of the psychosocial consequences experienced by children and adolescents living with congenital heart disease and their primary caregivers: a scoping review protocol
Source: Syst Rev. 2023 Jun 2;12:90. doi: 10.1186/s13643-023-02249-7 (PMC10239103; doi:10.1186/s13643-023-02249-7)
Supplement: Supplementary file 8 — Additional file 8. Instructions for completing data extraction form and sample data extraction form. [file 13643_2023_2249_MOESM8_ESM.docx]

**Additional File 8**

**Instructions for Completing Data Extraction and Sample Data Extraction Form**

**Instructions**

The data extraction form consists of six sections total. Complete the extraction independently. Sections one, five, and six must be completed. If you cannot find the data related to the question or component of the data extraction form, then write either missing or not applicable in the blank next to it. Section two, three, and four are designed in relation to the different objectives of the scoping review. At the end of section one you will be instructed to check all boxes that apply to the study in which you are extracting data from and then fill out the sections that corresponds to the box you have checked. If you did not check the box that corresponds to a specific section, then write not applicable on that section and move to the next section. Once extraction is completed by both extractors the data extracted by both extractors will be compared to ensure agreement or to identify discrepancies. One author’s paper data collection form will be used to record changes after consensus is reached by the two extractors in a different ink color. The table at the bottom of the form can be used to track consensus and disagreements.

**Sample Data Extraction form for Scoping Review**

| **Section 1: Citation Information, Study Characteristics, Study Participants and Objectives** | | |
| --- | --- | --- |
| **Name of Data Extractor:** | **Date of Data Extraction:** | |
| **Manuscript ID # in Endnote:** | **Manuscript ID in Covidence #:** | |
| **Citation Information** | | |
| **Title or Manuscript:** | | |
| **Author(s) *(include name, credentials, affiliations, and location):*** | | |
| **Journal Published in:** | | |
| **Volume:** | **Issue:** | |
| **Page numbers:** | **Year Published:** | |
| **Funding sources:** | **Other possible reporting biases:** | |
| **Study Design Characteristics** | | |
| **Study region(s) and country/countries:**  **Setting/Context *(e.g., outpatient, inpatient*):**  **Type of Study Design (*check one):***  **Quantitative**  **Qualitative**  **Mixed Methods**  **Multi-Method**  **Unclear**  **Study Methodology (e.g., randomized control study, phenomenology):**  **Study eligibility criteria (i.e., who qualified as participants for the study):**  **Recruitment and sampling procedure used (e.g., convenience, snowball):**  **Methods of data collection *(e.g., interviews, focus groups, charts, etc.):***  **Description of data collection method:**  **Enrollment start date:**  **Enrollment end date:**  **Is this an intervention study (*check one*):**  **Yes**  **No**  **Specific to intervention studies only:**  **Length of follow-up:**  **Allocation to intervention vs. comparator groups:** | | |
| **Study Participants** | | |
| **What study population do the participants belong to *(check all that apply):***  **Children or adolescents with CHD**  **Caregivers of children or adolescents with CHD**  **Number of total participants in the study:** | | |
| **Age of participants:** | **Gender distribution:** | |
| **Type(s) of CHD the child or adolescent has OR the child or adolescent of the caregiver has (*(include number for each type of CHD*):** | **Socio-economic status:** | |
| **Ethnicity distribution:** | **Surgical status of the child or adolescent OR the child or adolescent of the caregiver *(i.e., if they have had surgery and number of surgeries, have not had surgery):*** | |
| **Marital Status *(caregiver studies only):*** | **Other reported co-morbidities for the child or adolescent has OR the child or adolescent of the caregiver has:** | |
| **Other specific participant characteristics identified:** | | |
| **Scoping Review Objectives that the Study Describes** | | |
| **What does this study describe (*check all that apply and then fill out only the corresponding data extractions sections of the boxes you have checked*)?**  **Negative psychosocial consequence(s) experienced by children or adolescents with CHD (*proceed to section 2*)**  **Negative psychosocial consequence(s) of caregivers of children or adolescents with CHD (*proceed to section* 2)**  **Factor(s) influencing the development of negative psychosocial consequences in children or adolescents with CHD (*proceed to section 3*)**  **Factor(s) influencing the development of negative psychosocial consequences in caregivers of children or adolescents with CHD (*proceed to section 3*)**  **Intervention(s) to decrease the negative psychosocial consequences experienced by children or adolescents living with CHD (*proceed to section 4*)**  **Intervention(s) to decrease the negative psychosocial consequences experienced by caregivers of children or adolescents living with CHD (*proceed to section 4*)** | | |
| **Section 2: Negative psychosocial consequence(s) experienced by children or adolescents living with CHD and/or their caregivers** | | |
| **What study population do the participants belong to *(check all that apply):***  **Children or adolescents with CHD**  **Caregivers of children or adolescents with CHD**  **Type of Study Design (*check one and fill in corresponding table*):**  **Quantitative (*fill in Table 1*)**  **Qualitative (*fill in Table 2)***  **Mixed Methods (*fill in Table 1 and 2*)**  **Multi-Method (fill in Table 1 and 2)**  **Table 1: Quantitative studies or quantitative data from mixed method/multi-method studies** | | |
| **Psychosocial Consequence identified or measured, and Psychosocial scale used if any** | **Supporting data from the study (e.g., quantitative survey results, % of people reporting, or scale results)** | |
|  |  | |
| **Table 2: Qualitative studies or qualitative data from mixed method/multi-method studies** | | |
| **Psychosocial Consequence identified (Theme)** | **Brief description of the theme identified** | |
|  |  | |
| **Section 3: Factors identified that influence the development of psychosocial consequences experienced by children or adolescents living with CHD and/or their caregivers** | | |
| **What study population do the participants belong to *(check all that apply):***  **Children or adolescents with CHD**  **Caregivers of children or adolescents with CHD**  **Type of Study Design (check one and fill in corresponding table):**  **Quantitative (fill in Table 1)**  **Qualitative (fill in Table 2)**  **Mixed Methods (fill in Table 1 and 2)**  **Multi-Method (fill in Table 1 and 2)**  **Table 1: Quantitative studies or quantitative data from mixed method/multi-method studies** | | |
| **Factors identified that influence the development of psychosocial consequences** | | **Supporting data from study (e.g., survey results, % of people reporting)** |
|  | |  |
| **Table 2: qualitative studies or qualitative data from mixed method/multi-method studies** | | |
| **Factors identified that influence the development of psychosocial consequences (Theme)** | | **Brief description of the theme** |
|  | |  |
| **Section 4: Intervention(s) to decrease the negative psychosocial consequences experienced by children or adolescents living with CHD and/or their caregivers** | | |
| **What study population was the intervention designed for *(check all that apply):***  **Children or adolescents with CHD**  **Caregivers of children or adolescents with CHD**  **Who was involved in designing the intervention?**  **Description of the intervention to decrease negative psychosocial consequences:**  **What psychosocial consequence(s) is the intervention meant to decrease?**  **How was the intervention measured?**  **Is the intervention effective:**  **Yes**  **no** **unclear**  **What are the specific results of the intervention?**  **Adverse effects of intervention if any:**  **Other Comments:** | | |
| **Section 5: Missing Data** | | |
| **Was there any missing data?**  **Yes**  **no** **unclear**  **If yes how was the missing data handled?**  **For intervention studies was any of the participants loss to follow-up OR did any withdraw:**  **Yes  no unclear**  **If *yes* how many:**  **Reasons for loss to follow-up or participants withdrawing from intervention studies:** | | |
| **Section 6: Miscellaneous** | | |
| **Key conclusions from study authors:**  **Study limitations discussed by authors:**  **Correspondence required with study authors and why:**  **Other comments:** | | |

| **Consensus Tracking** |
| --- |
| **Date form reviewed for consensus:**  **Total number of discrepancies:**  **Consensus reached:**  **Yes**  **No**  **If *No* action taken:**  **Third person consulted to resolve discrepancy**  **Comments:**  **Study authors consulted to resolve discrepancy comments:**  **Comments:**  **Consensus reached:**  **Yes Date:**  **No**  **If *No* comment on what discrepancy needs to be documented in the review:** |
